# Supplementary material for: EnzymeHunter: Achieving fine-grained enzyme function prediction with a hierarchically aware contrastive learning framework
Source: Patterns (N Y). 2026 May 28;7(7):101567. doi: 10.1016/j.patter.2026.101567 (PMC13366525; doi:10.1016/j.patter.2026.101567)
Supplement: Document S1. Figures S1–S7 [file mmc1.pdf]

Patterns, Volume *7*

Supplemental information

EnzymeHunter: Achieving fine-grained enzyme function prediction with a hierarchically aware contrastive learning framework

Guoxin Cao, Jian Ouyang, Xiangyi Xiong, Changle Liu, Yi Zhang, Siqi Yang, Tieliu Shi, and Jun Wu

Supplemental information

EnzymeHunter: Achieving fine-grained enzyme function prediction with a hierarchically-aware contrastive learning framework

Guoxin Cao, Jian Ouyang, Xiangyi Xiong, Changle Liu, Yi Zhang, Siqi Yang, Tieliu Shi, Jun Wu

Supplemental Figures

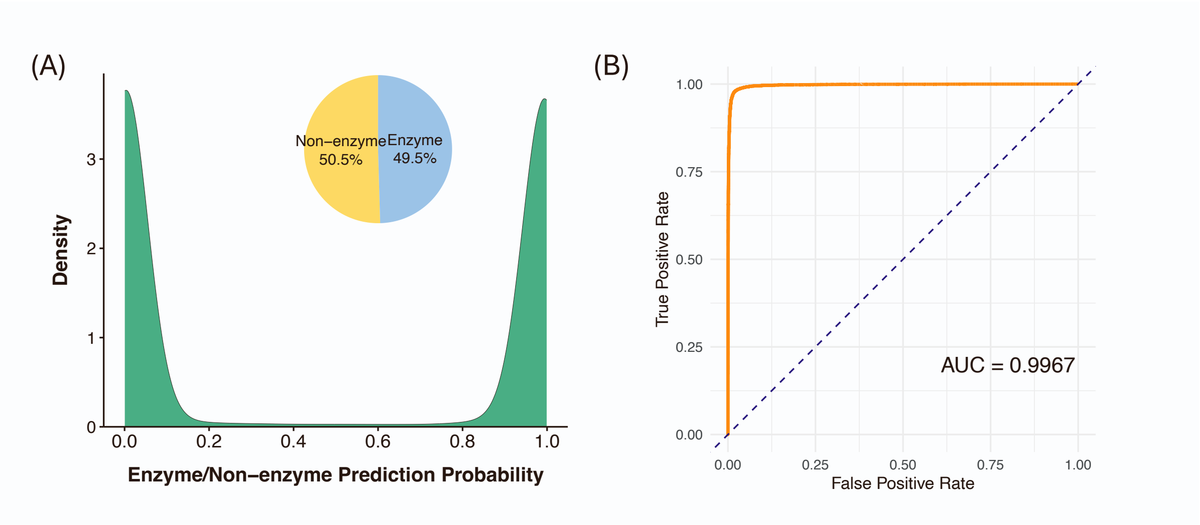

Figure S1. Bimodal distribution of enzyme prediction probabilities and performance in enzyme classification. (A) The histogram of predicted enzyme probabilities shows a clear bimodal distribution, with most proteins confidently classified as either non-enzymes (peak near 0) or enzymes (peak near 1). (B) Performance on the binary task of distinguishing enzymes from non-enzymes.

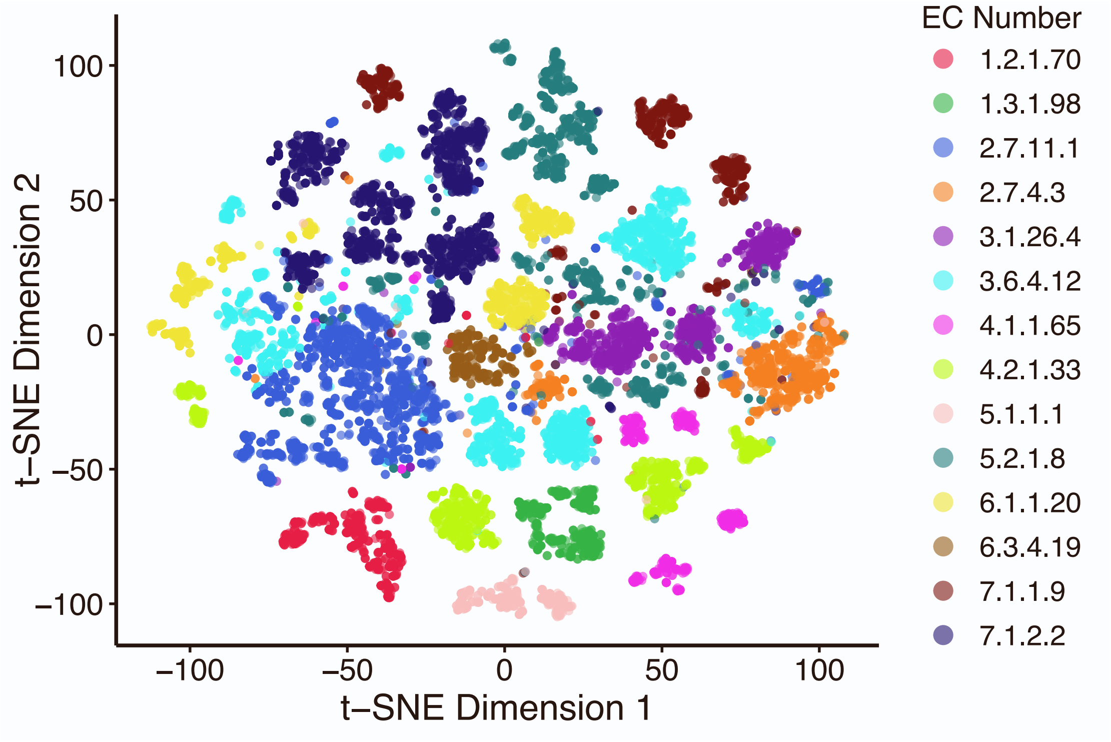

Figure S2. t-SNE projection of combined ESM2 and ResNet embedding representations. t-SNE projection of the concatenated 2560-dimensional ESM2 embeddings and 2048-dimensional ResNet embeddings.

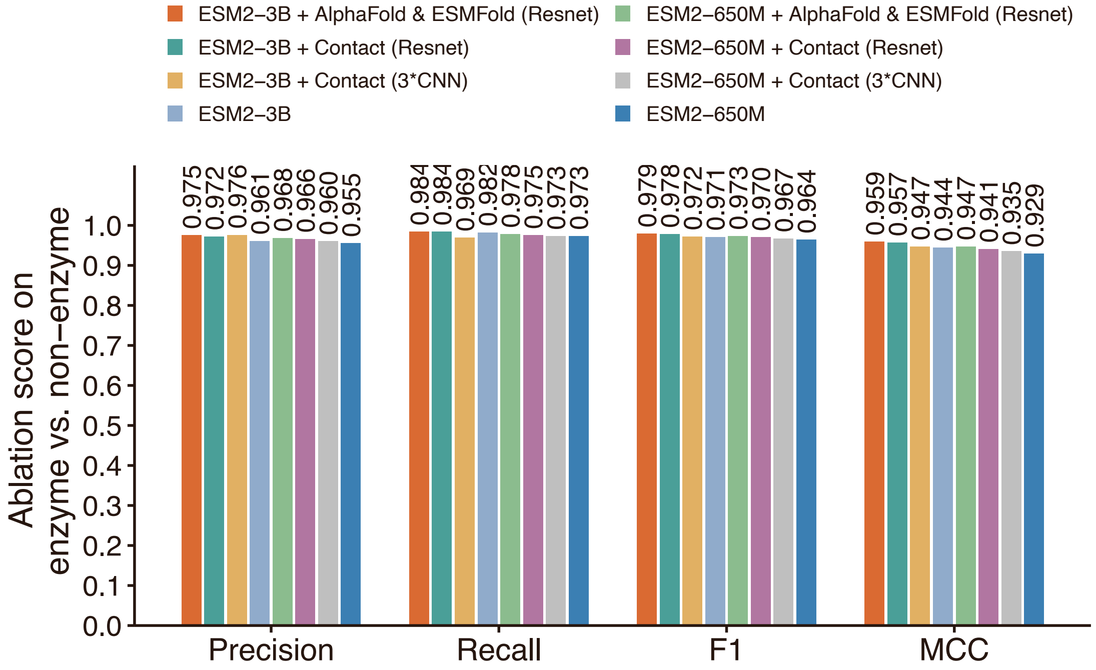

Figure S3. Performance comparison of different feature combinations on the enzyme vs. non-enzyme dataset. The model performance is evaluated using Precision, Recall, F1 score, and MCC.

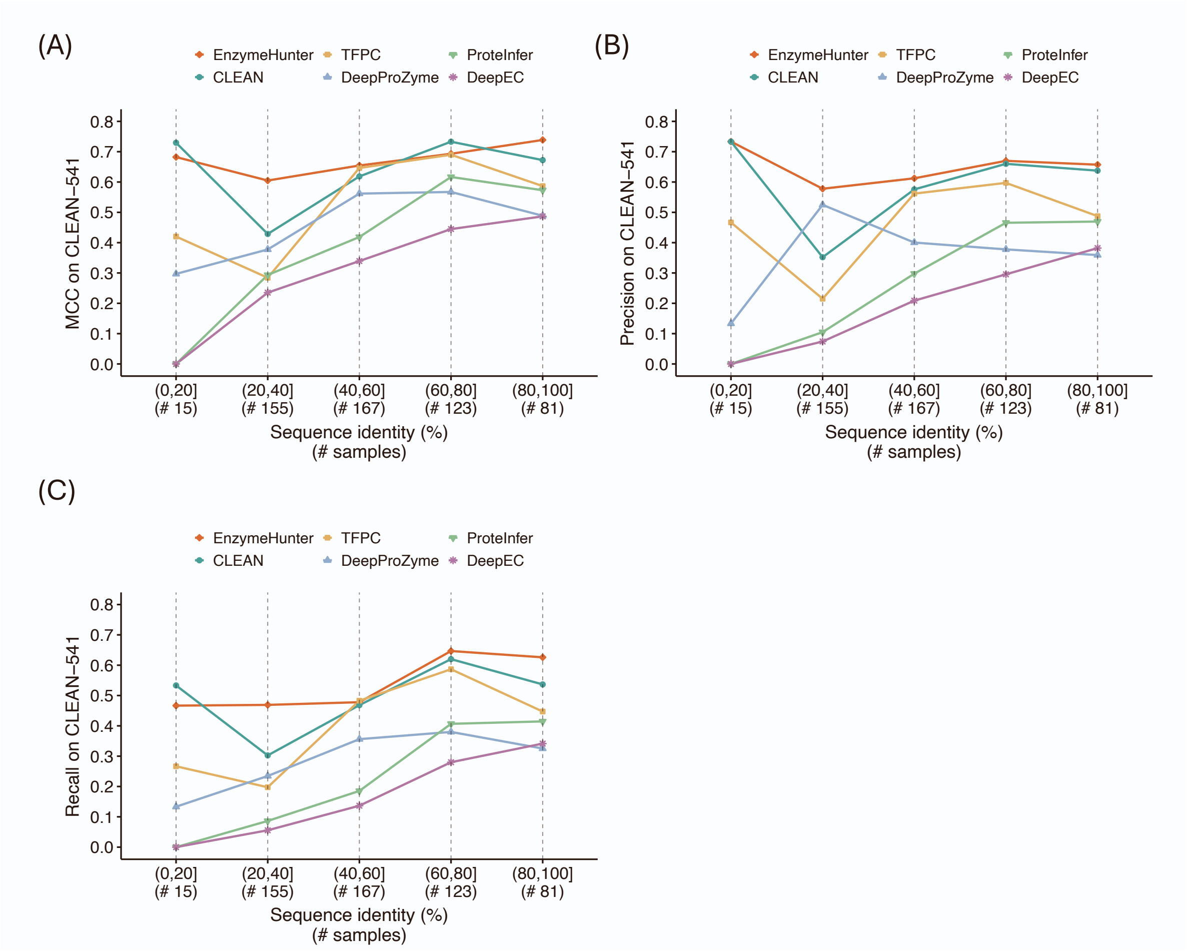

Figure S4. The performance results of the model on the Sequence identity in the CLEAN-541 dataset. (A) The results of the MCC metric on the CLEAN-541 dataset. (B) The results of the Precision metric on the CLEAN-541 dataset. (C) The results of the Recall metric on the CLEAN-541 dataset.

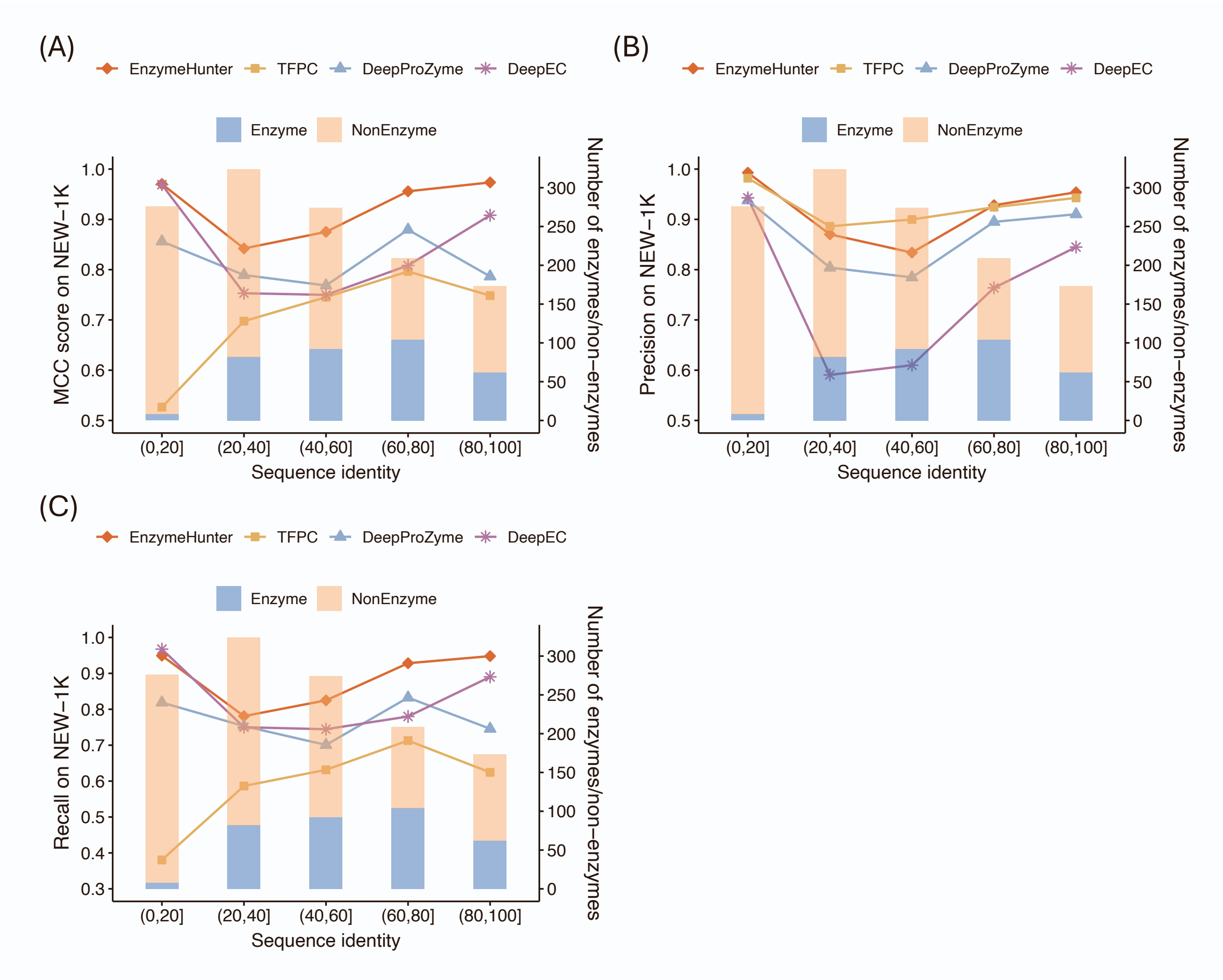

Figure S5. The model performance results on the NEW-1K dataset. (A) The results of the MCC metric on the NEW-1K dataset. (B) The results of the Precision metric on the NEW-1K dataset. (C) The results of the Recall metric on the NEW-1K dataset.

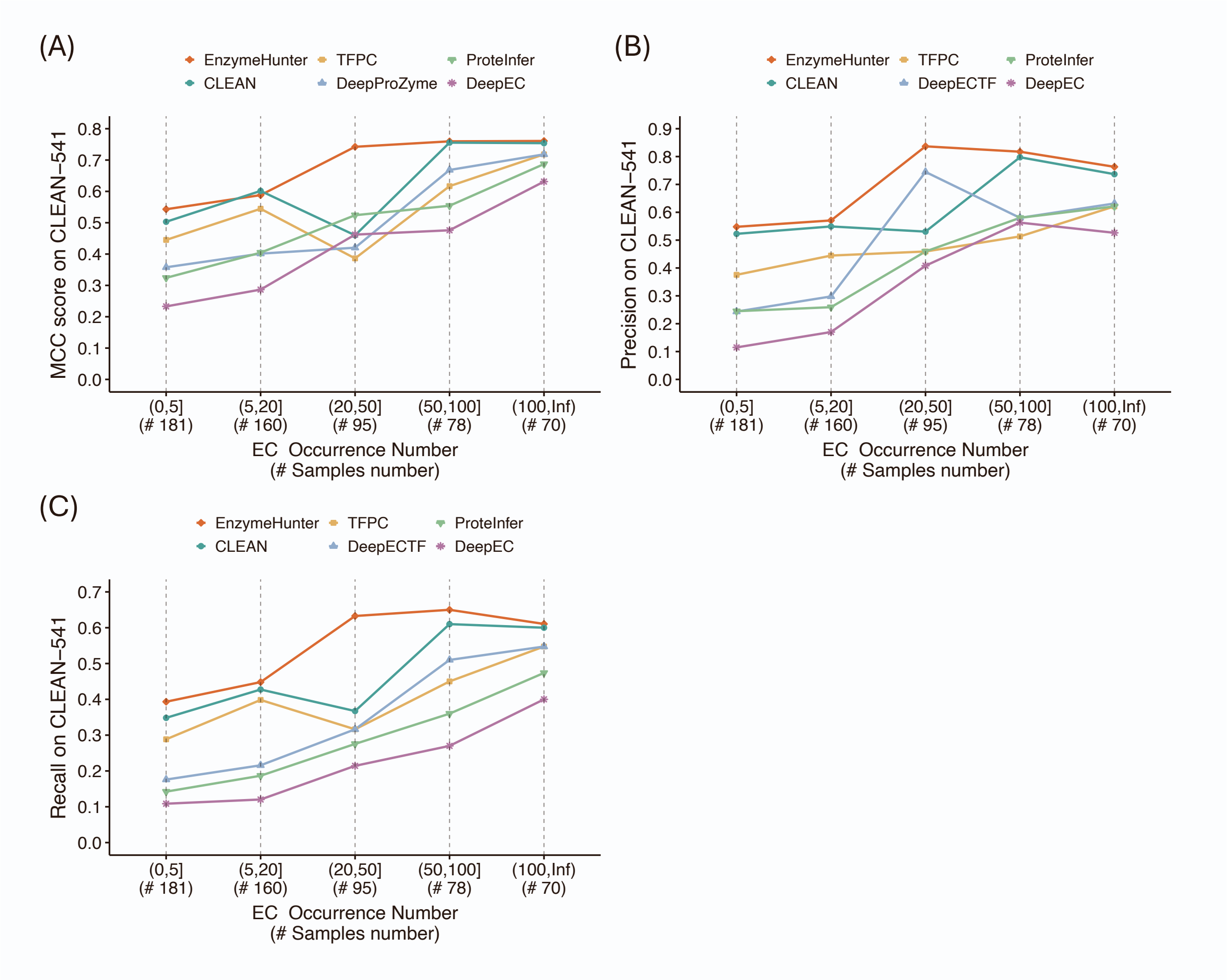

Figure S6. The performance results of the model on the EC Occurrence Number in the CLEAN-541 dataset. (A) The results of the MCC metric on the CLEAN-541 dataset. (B) The results of the Precision metric on the CLEAN-541 dataset. (C) The results of the Recall metric on the CLEAN-541 dataset.

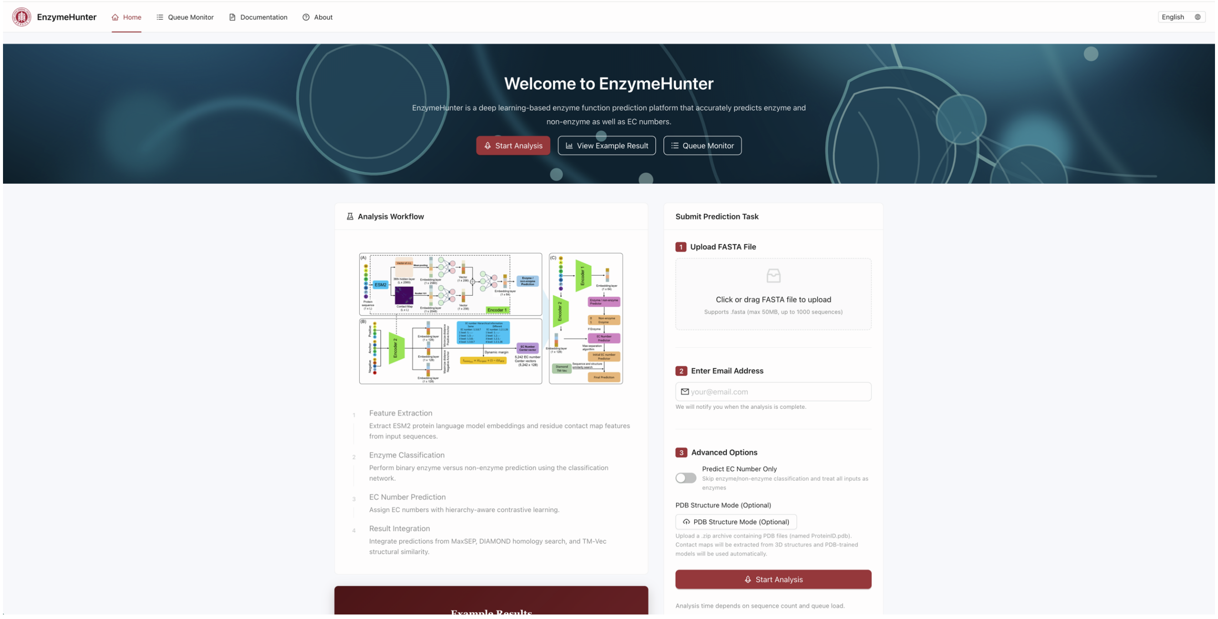

Figure S7. The web interface of EnzymeHunter (https://pathoatlas.com/EnzymeHunter). Users can upload FASTA files of amino acid sequences to obtain enzyme function prediction results.
