# Supplementary material for: EnzymeHunter: Achieving fine-grained enzyme function prediction with a hierarchically aware contrastive learning framework
Source: Patterns (N Y). 2026 May 28;7(7):101567. doi: 10.1016/j.patter.2026.101567 (PMC13366525; doi:10.1016/j.patter.2026.101567)
Supplement: Document S2. Article plus supplemental information [file mmc2.pdf]

Article

Patterns

EnzymeHunter: Achieving fine-grained enzyme function prediction with a hierarchically aware contrastive learning framework

Graphical abstract

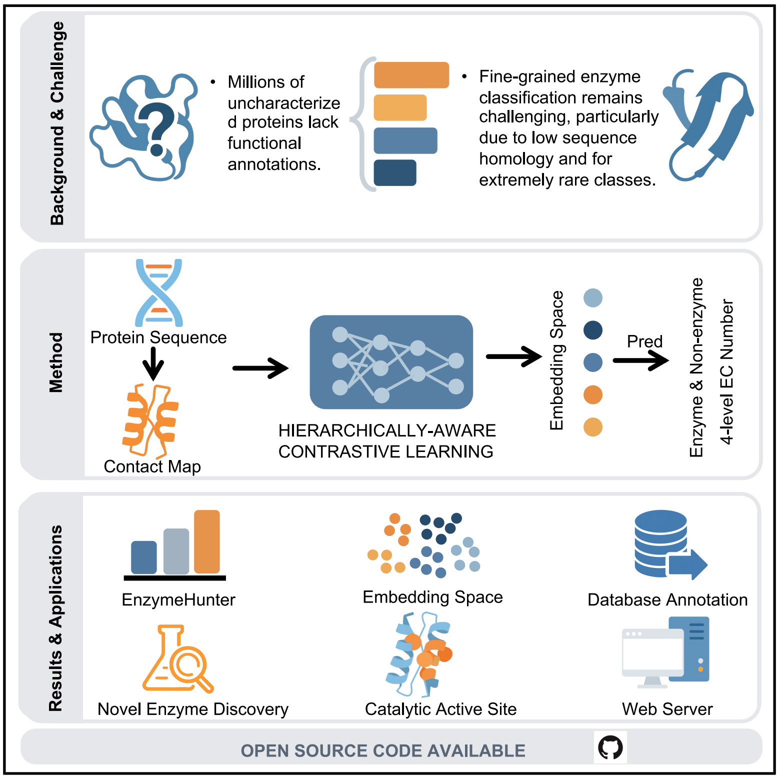

Authors

Guoxin Cao, Jian Ouyang, Xiangyi Xiong, ..., Siqi Yang, Tieliu Shi, Jun Wu

Correspondence

tieliushi@yahoo.com (T.S.), jwu@bio.ecnu.edu.cn (J.W.)

In brief

EnzymeHunter is a deep-learning framework for fine-grained enzyme function annotation based on hierarchically aware contrastive learning. It captures subtle catalytic differences, achieves state-of-the-art performance in low-homology scenarios, and accurately predicts rare enzyme classes. At the proteome scale, it can reveal novel enzymatic functions and provide interpretable insights into catalytic mechanisms.

Highlights

- Hierarchically aware contrastive learning for EC prediction
- Accurate prediction in low-homology and rare enzyme classes
- Proteome-scale annotation reveals novel enzyme functions
- Interpretable predictions and an accessible web server

Cao et al., 2026, Patterns 7, 101567
July 10, 2026 © 2026 The Authors. Published by Elsevier Inc.
https://doi.org/10.1016/j.patter.2026.101567

CellPress

Patterns

CellPress
OPEN ACCESS

Article

EnzymeHunter: Achieving fine-grained enzyme function prediction with a hierarchically aware contrastive learning framework

Guoxin Cao,[1,6] Jian Ouyang,[1,2,6] Xiangyi Xiong,[1,6] Changle Liu,[1] Yi Zhang,[5] Siqi Yang,[1] Tieliu Shi,[1,3,4,*] and Jun Wu[1,4,7,*]
[1]Center for Bioinformatics and Computational Biology, and The Institute of Biomedical Sciences, School of Life Sciences, East China Normal University, Dongchuan Road 500, Shanghai 200241, China
[2]Henan International Joint Laboratory of Infection and Immunity, Henan Key Laboratory of Critical Care Medicine, Department of Emergency Medicine, The First Affiliated Hospital, Zhengzhou University, East Jianshe Road No. 1, Zhengzhou 450052, China
[3]Key Laboratory of Advanced Theory and Application in Statistics and Data Science -Ministry of Education, School of Statistics, East China Normal University, Zhongshan North Road 3663, Shanghai 200062, China
[4]Shanghai Institute of Wildlife Epidemics, East China Normal University, Dongchuan Road 500, Shanghai 200062, China
[5]School of Statistics, East China Normal University, Zhongshan North Road 3663, Shanghai 200062, China
[6]These authors contributed equally
[7]Lead contact
*Correspondence: tieliushi@yahoo.com (T.S.), jwu@bio.ecnu.edu.cn (J.W.)
https://doi.org/10.1016/j.patter.2026.101567

THE BIGGER PICTURE Understanding enzyme function is essential for interpreting genomes and enabling advances in biotechnology and medicine. Most proteins, however, remain uncharacterized, and distinguishing subtle functional differences between enzymes remains challenging, especially when sequence similarity is low. Recent computational approaches have improved enzyme annotation, but many struggle to achieve fine-grained functional resolution or to generalize across diverse enzyme families. EnzymeHunter provides a unified framework that integrates sequence and structural information while leveraging the hierarchical organization of enzyme functions. By enabling more precise and scalable enzyme annotation, EnzymeHunter helps researchers better explore the functional diversity of proteins and accelerates discoveries across genomics, biotechnology, and synthetic biology.

SUMMARY

Accurate enzyme function annotation is a grand challenge due to the vast number of uncharacterized proteins and the difficulty of distinguishing subtle functions. We introduce EnzymeHunter, a deep-learning framework that achieves fine-grained prediction via a hierarchically aware contrastive learning strategy. By integrating sequence and structural information and using the Enzyme Commission (EC) hierarchy to guide its loss function, our model learns a functionally coherent embedding space where distances reflect precise levels of catalytic similarity. EnzymeHunter significantly outperforms state-of-the-art models, particularly in challenging scenarios, achieving fine-grained precision down to the fourth EC level, maintaining robust performance in low-homology cases, and accurately predicting rare enzyme classes. In a proteome-wide application to *Thermus thermophilus*, EnzymeHunter discovered novel catalytic functions, one of which was subsequently validated by an independent UniProt update. Furthermore, our model is interpretable, with predictions guided by learned attention on mechanistically critical functional sites.

INTRODUCTION

Enzymes mediate the vast majority of biochemical reactions essential for life, driving critical processes such as protein synthesis, energy metabolism, and signal transduction.[1,2] The advent of high-throughput sequencing has created a vast disparity between the volume of sequence data and our capacity for functional validation. Public databases like UniProt now list over 250 million sequences, yet only a tiny fraction (~0.2%) has been manually reviewed (Swiss-Prot), and even fewer have

This is an open access article under the CC BY license (http://creativecommons.org/licenses/by/4.0/).

direct experimental evidence.[3] This functional annotation gap is especially pronounced in the microbial universe, where up to 70% of proteins constitute “functional dark matter.”[4–6] This vast sea of uncharacterized proteins represents a major bottleneck, impeding our fundamental understanding of biology and the discovery of novel biocatalysts.

To systematically classify enzymatic functions, the Enzyme Commission (EC) number system provides a hierarchical framework.[7,8] However, as experimental characterization cannot keep pace with the data deluge, the field has increasingly turned to computational methods. While traditional homology-based approaches are powerful, their efficacy diminishes significantly when annotating novel enzymes with low sequence identity to characterized proteins.[9,10] This limitation has spurred a paradigm shift toward deep learning.[11–13] A pivotal driver of recent progress has been the application of large-scale protein language models (PLMs) such as ESM2,[14] which learn rich, context-aware representations from massive sequence data and have proven highly effective across a wide range of protein prediction tasks. This evolution has proceeded along two main trajectories: sequence-based and structure-based approaches. Sequence-centric methods saw early success with convolutional neural networks (DeepEC) and later more powerful architectures such as transformers (DeepECtransformer).[15,16] The integration of PLMs in methods like CLEAN[17] and ESM-Ezy[18] marked a significant leap forward, with further innovations such as contrastive learning being introduced to tackle data imbalance. Concurrently, structure-based methods like TopEC emerged,[19] utilizing 3D graph neural networks on experimentally determined or predicted protein structures to capture the geometric and chemical features of the active site.

Despite these remarkable advances, several critical challenges persist. Sequence-based models often struggle with low-homology cases where functional signals are not conserved in the primary sequence.[20–22] Conversely, structure-based models can be limited by the availability of high-quality 3D structures and may not fully capture the dynamic or evolutionary context embedded in sequences. Furthermore, for both types of models, achieving the fine-grained precision required to differentiate between closely related EC sub-subclasses (level 4), a distinction that is often vital for biological understanding, is challenging. Finally, the inherent data imbalance of functional annotations continues to bias models toward common enzymes, and the “black-box” nature of many deep-learning models can obscure the biochemical basis of their predictions.

To overcome these limitations and unify the strengths of both approaches, we developed EnzymeHunter. Our framework is based on a hierarchically aware contrastive learning framework that synergistically integrates deep sequence embeddings with predicted structural information. Unlike conventional methods, our loss function is explicitly informed by the EC hierarchy, training the model to learn a functionally coherent embedding space where distances reflect precise levels of catalytic similarity. This unique strategy directly addresses the challenges of fine-grained prediction and data imbalance. In this work, we demonstrate that EnzymeHunter not only establishes a new state of the art in performance but also shows exceptional capability in annotating enzymes with low homology, resolving precise subclass-level functions, and illuminating the functions of previously uncharacterized proteins in a real-world proteome.

RESULTS

Architecture of EnzymeHunter

The architecture of EnzymeHunter is designed to synergistically integrate information from both protein sequence and predicted structure, with both feature modalities being generated efficiently from a single input sequence using the state-of-the-art ESM-2 protein language model (Figure 1A). The overall architecture consists of a unified feature-extraction backbone followed by two specialized task-specific heads.

The foundation of our model is this dual-modality feature backbone. To generate the sequence representation, we extract the output from the final (36th) hidden layer of the ESM-2 model and apply mean pooling across the sequence length, yielding a fixed-size, 2,560-dimensional global feature vector. Concurrently, to generate the structure-aware representation, we leverage ESM-2’s ability to predict residue-residue contacts. An $L \times L$ contact matrix is first obtained and then binarized using a probability threshold of 0.8 to retain only high-confidence interactions. This binary map, which provides key structural insights, is then expanded into a three-channel matrix to meet the input requirements of a pre-trained ResNet101 model.[23] By processing this map with ResNet101, which is highly effective at capturing multiscale hierarchical features, we produce a 2,048-dimensional vector that serves as our structure-aware representation.

The extracted sequence and structure features are then channeled into two distinct, task-specific prediction heads.

- Enzyme vs. non-enzyme classification head: for the binary classification task, the 2,560-dimensional sequence vector and the 2,048-dimensional structure vector are each independently projected to a 256-dimensional space via separate fully connected (FC) layers. These are then concatenated to form a 512-dimensional fused feature vector, which is further processed through an FC layer to 64 dimensions before being fed into the final sigmoid classification layer (Figure 1A).
- EC number prediction head: for the more complex EC number prediction task, a dedicated head is employed to map proteins into a 128-dimensional, functionally meaningful embedding space. To generate this embedding, the sequence and structural vectors are first projected to 512 dimensions each, concatenated into a 1,024-dimensional vector, and subsequently passed through a final FC layer. The training of this embedding space is optimized using a protein-protein triplet contrastive learning objective, the details of which are illustrated in Figure 1B. Specifically, a hierarchically aware loss (H-loss) function is incorporated to capture hierarchical relationships among enzyme annotations (see methods). The goal is to structure the latent space such that proteins with identical EC numbers are clustered together, while proteins with different EC numbers are pushed apart. During training, triplets are constructed from the training set: for a given

CellPress
OPEN ACCESS

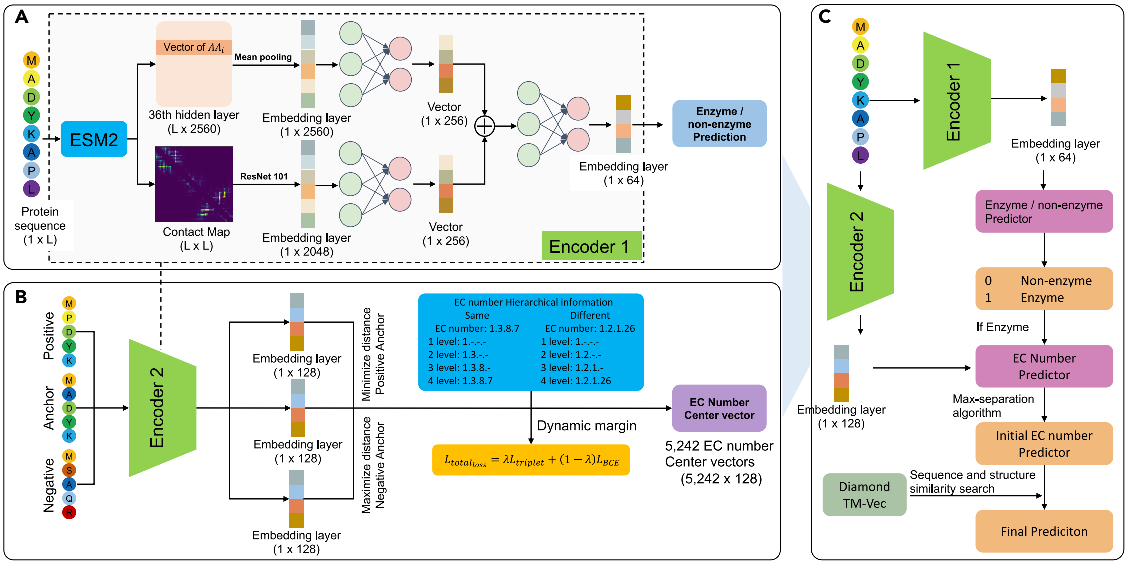

Figure 1. Architecture and workflow of the EnzymeHunter model

(A) The enzyme vs. non-enzyme classification head fuses the sequence and structural representations into a 512-dimensional vector, which is processed through fully connected layers to yield a binary prediction.

(B) The EC number prediction head employs a more complex architecture, creating a 1,024-dimensional fused vector that is projected into a 128-dimensional embedding space. This space is optimized via a hierarchically aware triplet contrastive loss to ensure functional similarity is reflected by geometric proximity.

(C) The overall workflow illustrates the complete two-stage prediction process: an input sequence is first classified as an enzyme or non-enzyme. If identified as an enzyme, it is then passed to the EC number prediction model to generate a precise, four-digit functional annotation.

anchor protein, a positive sample is another protein sharing the same full EC number, and a negative sample is a protein with a different EC number, selected via a hard negative mining strategy. This scheme forces the model to learn the fine-grained distinctions necessary for high-resolution functional prediction.

The complete two-stage prediction process, which integrates both heads, is illustrated in the overall workflow in Figure 1C.

EnzymeHunter outperforms state-of-the-art prediction models

To establish the performance of our model, we conducted a comprehensive benchmark comparison of EnzymeHunter against five state-of-the-art methods: DeepEC,[15] DeepECTF (short for DeepECtransformer),[16] CLEAN,[17] ProteInfer,[24] and TFPC.[25] Across a suite of challenging datasets designed to test distinct aspects of performance, EnzymeHunter consistently demonstrated superior accuracy and reliability, outperforming the compared methods.

A foundational design choice in EnzymeHunter was the use of a dedicated binary classifier to first distinguish enzymes from non-enzymes. This strategy proved highly effective, as EnzymeHunter achieved the best performance with an F1 score and area under the curve on this initial task (Figures 2A, S1A, and S1B). This result significantly outperforms competing models capable of this distinction, such as DeepEC (one-sided paired bootstrap test, $p < 0.001$) and DeepECTF (one-sided paired bootstrap test, $p < 0.001$), which hovered around an F1 score of 0.8. This strongly validates our hypothesis that decoupling the binary classification from the more complex EC number prediction leads to a more robust initial filtering of sequences. Moving to the specific and more challenging task of EC number prediction, EnzymeHunter established its state-of-the-art status on the merged benchmark dataset CLEAN-541. The results showed that the proposed model achieved a weighted F1 score of 0.568, marking a substantial 13.83% improvement over the previous leading method, CLEAN (one-sided paired bootstrap test, $p < 0.001$) (Figure 2B).

We further tested this entire two-stage pipeline on our novel, temporally disjoint New-1K dataset, which assesses the model's end-to-end capability on the most recent data. Here again, EnzymeHunter demonstrated superior performance, achieving a weighted F1 score of 0.894 and significantly outperforming all baseline models (one-sided paired bootstrap test, $p < 0.001$ for all comparisons) (Figure 2C). All these comparison results showed that EnzymeHunter not only achieved higher scores but also showed a well-balanced performance with significant gains in both precision and recall, indicating enhanced sensitivity and reliability.

To elucidate the mechanistic basis for this superior predictive performance, we visualized the raw, unprocessed feature vectors with the final 128-dimensional embeddings produced by EnzymeHunter (Figures 2D and S2). While the initial features show unstructured and overlapping clusters, the embeddings transformed by EnzymeHunter exhibit clear, well-separated clusters where proteins of the same EC class are tightly grouped. This visualization provides qualitative evidence that our model successfully learns to organize the feature space in a functionally meaningful way, providing a clear rationale for its superior performance in downstream prediction tasks.

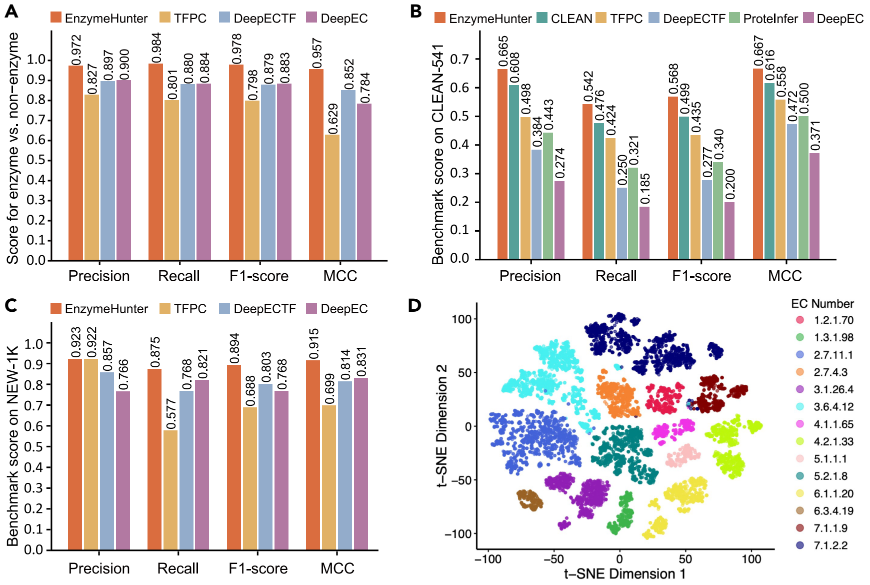

Figure 2. Comparative performance of EnzymeHunter against state-of-the-art methods

(A) Performance on the binary task of distinguishing enzymes from non-enzymes.

(B) Performance on the merged CLEAN-541 benchmark from the CLEAN study. Metrics shown are weighted precision, recall, F1 score, and MCC.

(C) End-to-end predictive performance on the novel New-1K temporal test set, which assesses both classification and subsequent EC number prediction.

(D) t-Distributed stochastic neighbor-embedding projection of the final 128-dimensional embeddings learned by EnzymeHunter.

Ablation studies and component contributions

To comprehensively evaluate the contribution of each component within our framework, we conducted extensive ablation studies across both the binary enzyme-vs.-non-enzyme classification and the multilabel EC number annotation tasks (see methods). On the fundamental classification task (Figure S3), scaling the protein language model from ESM2-650M to ESM2-3B and incorporating structural information were associated with higher performance. These components also contributed to the primary EC annotation task on the CLEAN-541 dataset (Figure 3A). Starting with sequence-only baselines, scaling from ESM2-650M to ESM2-3B increased the F1 score from 0.471 to 0.51. Incorporating structural information further enhanced performance. Notably, for processing 2D structural representations, we found that a ResNet-based architecture yielded higher performance than the standard convolutional neural network (CNN); coupling this ResNet-processed contact map with ESM2-3B increased the F1 score to 0.557 and Matthew's correlation coefficient (MCC) to 0.653. Crucially, removing the H loss markedly degraded these results (F1 score dropped from 0.557 to 0.519 for the contact map version and from 0.551 to 0.533 for the 3D version), underscoring its necessity for accurately modeling the EC hierarchy. Finally, integrating retrieval-based evidence via DIAMOND and TM-Vec consistently yielded the best results, demonstrating the strong efficacy of combining learned representations with similarity-based signals.

To further optimize the critical H-loss component, we evaluated multiple weight distributions across the four EC levels (Figure 3B). We found that a configuration of [0.4, 0.30, 0.2, 0.1] maximized annotation performance. This suggests that moderately emphasizing higher-level, more general EC information while retaining a focus on specific sublevel features creates a highly effective embedding space for hierarchical prediction.

As shown in Figure 3A, while the model utilizing explicit 3D structural representations (retrieved from AlphaFoldDB[26] or predicted using ESMFold[27]) combined with retrieval modules achieved the highest observed accuracy (F1 score = 0.570, MCC = 0.684), its generation and processing are computationally intensive. Conversely, the model utilizing lightweight contact maps with retrieval modules achieved highly competitive performance (F1 score = 0.568, MCC = 0.667). Therefore, we provide both a 3D-structure-based version and a contact-map-based version of EnzymeHunter to accommodate different computational budgets. Due to this minimal performance gap and significant computational savings, unless otherwise stated, the subsequent analyses in this article are based on the contact-map version for efficiency and reproducibility.

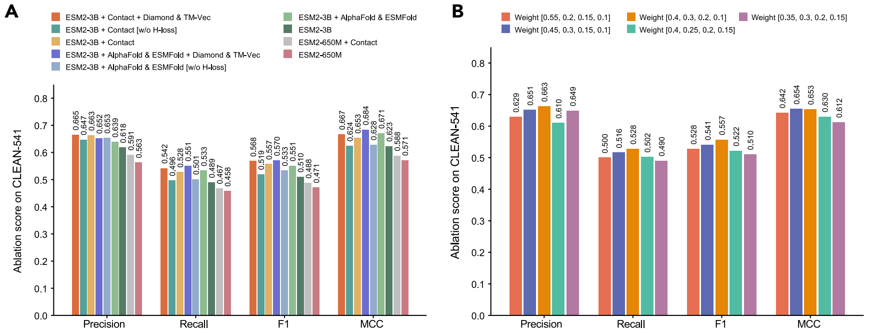

Figure 3. Ablation studies and hyperparameter optimization on the CLEAN-541 dataset

(A) Component ablation study. Performance comparison across model configurations varying the protein language model, structural input (2D contact map vs. 3D structure), H loss, and the retrieval module.

(B) H-loss weight optimization. Evaluation of model performance using different weight distributions for the four levels of the EC hierarchy.

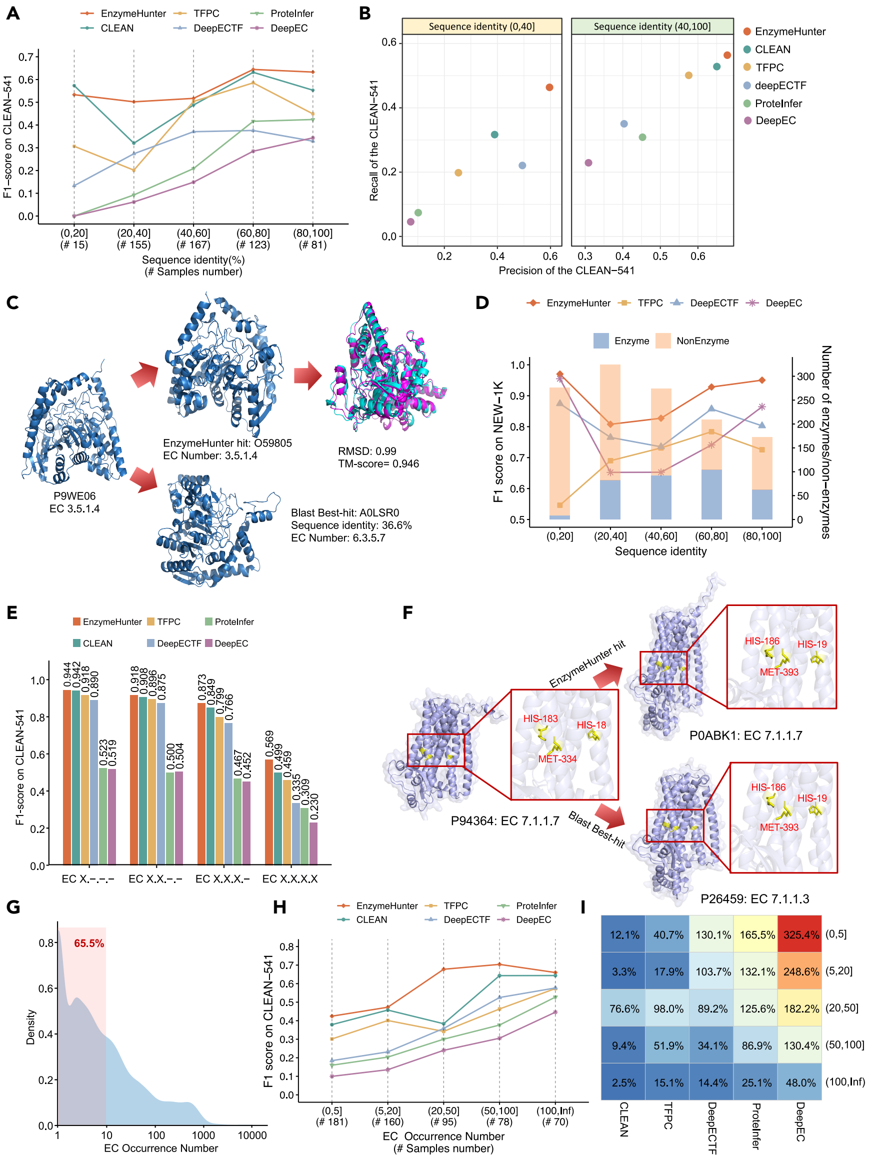

Figure 4. EnzymeHunter demonstrates robust and precise performance across key challenges

(A) Performance in low-homology scenarios on the CLEAN-541 enzyme set. F1 scores are stratified by sequence identity. EnzymeHunter's advantage is most pronounced in the challenging 20%–40% identity range.

(B) Precision-recall balance for low-homology (<40%) prediction. The position of EnzymeHunter relative to other models indicates a superior balance of precision and recall.

(C) Case study: overcoming misleading sequence homology. For query P9WEP6 (EC 3.5.1.4), EnzymeHunter correctly identifies the function via a high-confidence structural homolog (top, O59805), bypassing the incorrect top BLAST hit (bottom, A0LSR0).

(D) Performance on the comprehensive New-1K dataset. High F1 scores in low-homology bins are driven by the two-stage pipeline's effective filtering of non-enzymatic sequences.

(E) Fine-grained predictive precision across the EC hierarchy. F1 scores at each of the four EC levels. The performance gap widens at the most specific level 4, where EnzymeHunter shows a 14.7% gain over the next-best model.

(F) Contrastive learning enables fine-grained prediction. Case study of P94364 (left, EC 7.1.1.7). The model correctly predicts the function as 7.1.1.7. An analysis of the learned embedding space shows the model correctly pairs the query with P0ABK1 (bottom), which shares the correct function. This learned functional pairing bypasses the misleading top BLAST hit (top).

(G) Long-tail distribution of enzyme classes. A density plot of EC number frequencies in the training data illustrates that a vast majority of enzyme classes are rare.

(H) Superior performance on rare enzyme classes. F1 scores stratified by EC number frequency. EnzymeHunter shows a significant performance advantage in the lowest-frequency bins (≤5 occurrences).

(I) Comprehensive performance advantage across the frequency spectrum. A heatmap visualizes the percentage F1-score improvement of EnzymeHunter over each baseline model in different frequency bins.

Addressing key challenges in functional annotation

While benchmark performance provides a crucial baseline, a model's true utility is defined by its performance on the most difficult cases that plague computational enzymology. These include the challenge of the difficulty of annotating novel enzymes in the low-homology "twilight zone," achieving fine-grained functional resolution at the substrate-specific fourth EC level, and the persistent problem of accurately predicting rare functions from imbalanced datasets. We next evaluated EnzymeHunter's performance against these specific long-standing challenges.

First, we tested the model's robustness in low-sequence-homology scenarios, a "twilight zone" (<40% identity) where homology-based methods often fail. On the CLEAN-541 enzyme-only dataset, EnzymeHunter's advantage was particularly marked (Figures 4A, 4B, and S4). In the critical 20%–40% identity range, its F1 score of 0.502 represents a 56.9% improvement over the state-of-the-art model, CLEAN. This success stems from the model's ability to identify structural homologs even when sequence similarity is low. A compelling example is the amidase P9WEP6 (EC 3.5.1.4), detailed in Figure 4C. Its best blast hit (A0LSR0, 36.6% identity) is functionally incorrect (EC 6.3.5.7). In contrast, EnzymeHunter bypasses this misleading sequence signal and correctly predicts EC 3.5.1.4 by identifying O59805 as a structurally homologous match (template modeling [TM] score = 0.946). This structure-driven approach is the key to its superior performance. We observed similar robust performance on the comprehensive New-1K dataset, where our two-stage pipeline excelled at filtering out non-enzymatic sequences in low-homology bins (Figures 4D and S5).

Next, we assessed the model's ability to achieve fine-grained predictive precision across the EC hierarchy. While most top

models perform well at broader classifications, the key challenge lies in distinguishing functions at the substrate-specific fourth level. The results, shown in Figure 4E, reveal a clear performance divergence at this most granular level. EnzymeHunter achieves a remarkable level 4 F1 score of 0.569, representing a substantial 14.7% improvement over the previous state-of-the-art model, CLEAN. This capacity to resolve even subtle functional distinctions is mainly attributed to our hierarchically aware contrastive learning framework, as illustrated for P94364 (EC 7.1.1.7) in Figure 4F. While a standard BLAST search is misleading, pointing to a protein with an incorrect function (P26459, EC 7.1.1.3), EnzymeHunter directly predicts the correct EC number, 7.1.1.7. Inspection of the learned embedding space reveals that the nearest neighbor to P94364 is P0ABK1, an enzyme with the correct 7.1.1.7 function. A retrospective structural alignment confirms the biological validity of this representation by establishing that the two enzymes are genuine structural homologs (TM score = 0.935). This demonstrates an advanced level of learning, as the model correctly groups these proteins by their overall functional architecture, even though their specific binding-site residue positions differ (e.g., His18 in the query vs. His19 in the match), enabling it to deliver the accurate, fourth-level predictions essential for applications like metabolic engineering.

Finally, we addressed the challenge of overcoming data imbalance to predict rare enzyme functions. The long-tail distribution of EC numbers in training data often biases models toward common classes, a problem many methods circumvent by simply excluding rare data (Figure 4G). We confronted this issue directly by evaluating performance on classes stratified by their occurrence frequency. EnzymeHunter demonstrates a dramatic performance advantage for the most under-represented classes (Figures 4H and S6). For extremely rare EC numbers ($\leq$5 occurrences), the observed relative increases in F1 score were 325% over DeepEC and 41% over TFPC. This enhanced performance is attributable to our contrastive learning framework, which uses hard negative mining and data augmentation for singletons to explicitly learn discriminative features for these rare classes. Notably, the non-monotonic performance, such as the slight drop in the >100 bin, is likely attributable to the trade-off whereby the learning challenge posed by the high intra-class diversity of these broad enzyme families outweighs the statistical benefit of a larger sample size. As summarized in the heatmap (Figure 4I), this results in a remarkably stable performance profile across the entire frequency spectrum, demonstrating that high accuracy on rare classes is achievable without discarding valuable data.

Resolving incomplete and multifunctional enzyme annotations

Beyond benchmark performance, we also present qualitative case studies to illustrate our model's practical utility on complex, real-world annotation challenges. As of April 2025, for instance, of the nearly 280,000 reviewed enzymes in the UniProt database, a significant portion either lack complete functional annotation (16.5%) or are known to be multifunctional (6.8%). These ambiguities present a major challenge for computational tools. To demonstrate EnzymeHunter's practical value in addressing these specific issues, we present the following case studies.

Our first case study demonstrates EnzymeHunter's ability to complete a vague, top-level annotation. We examined an apoptosis-inducing factor homolog (Q54NS8), which was annotated in UniProt only as a general oxidoreductase (EC 1.-.-.-). EnzymeHunter assigned a precise function, predicting the complete EC number 1.6.5.9 (NADH: ubiquinone oxidoreductase). This prediction was strongly substantiated: the protein contains the characteristic Pyr_redox_2 domain, and despite low sequence identity (27.8%), its predicted structure shows remarkable similarity (TM score = 0.802) to a confirmed EC 1.6.5.9 enzyme, P44856 (Figure 5A).

Next, we addressed a partially annotated enzyme, the UDP-glycosyltransferase UGT71A15 (D3THI6), listed as EC 2.4.1.-. Our model successfully resolved this ambiguity by predicting the complete EC number, 2.4.1.91. This high-confidence assignment was validated by multiple lines of evidence: the presence of the required UDPGT domain (PF00201), the conservation of key catalytic residues, and exceptional structural similarity (TM score = 0.953) to a known EC 2.4.1.91 enzyme, Q2V6K0 (Figure 5B). Together, these cases underscore our model's reliability in completing partial EC numbers.

Our final and most challenging case addresses multifunctionality, a known blind spot for many prediction tools. We analyzed the pinene synthase R9QMQ9, which is correctly annotated with two distinct activities (EC 4.2.3.119 and 4.2.3.120). As shown in the summary table (Figure 5C), EnzymeHunter was the only model to correctly predict both functions. The basis for this success is revealed by a direct comparison of the top sequence and embedding space hits (Figure 5D). Notably, this prediction represents a highly confident "dual-confirmation" scenario. First, the primary embedding-based maximum separation independently and successfully generated the correct multifunctional candidate EC numbers (4.2.3.119 and 4.2.3.120). The robustness of this embedding space is further highlighted when compared to sequence-based retrieval. The top BLAST hit, Q84KL4, is a compelling lure, sharing extremely high sequence (93.7% identity) and structural (TM score = 0.955) similarity with the query. However, it is functionally inaccurate, annotated with a related but incorrect EC number (4.2.3.111). In contrast, an analysis of EnzymeHunter's learned embedding space identifies Q6XDB5 as the nearest neighbor. While this protein has lower sequence identity (77%), it is a strong structural homolog (TM score = 0.937) and, critically, a true functional analog, as it is also a multifunctional enzyme.

Proteome-wide annotation of *Thermus thermophilus* reveals and validates novel catalytic functions

To demonstrate EnzymeHunter's utility in a real-world, large-scale discovery context, we applied it to the entire proteome of the thermophilic bacterium *Thermus thermophilus* HB8 (downloaded February 2025). This organism is a source of valuable thermostable enzymes, yet the function of a significant portion of its 2,227 proteins remains unreviewed. Of these, 549 proteins carry existing EC annotations, which are distributed across the seven top-level functional classes as shown in Figure 6A, with transferases (EC 2) being the most prevalent. After filtering for proteins present in our training set, we were left with a test proteome of 1,795 sequences for our analysis.

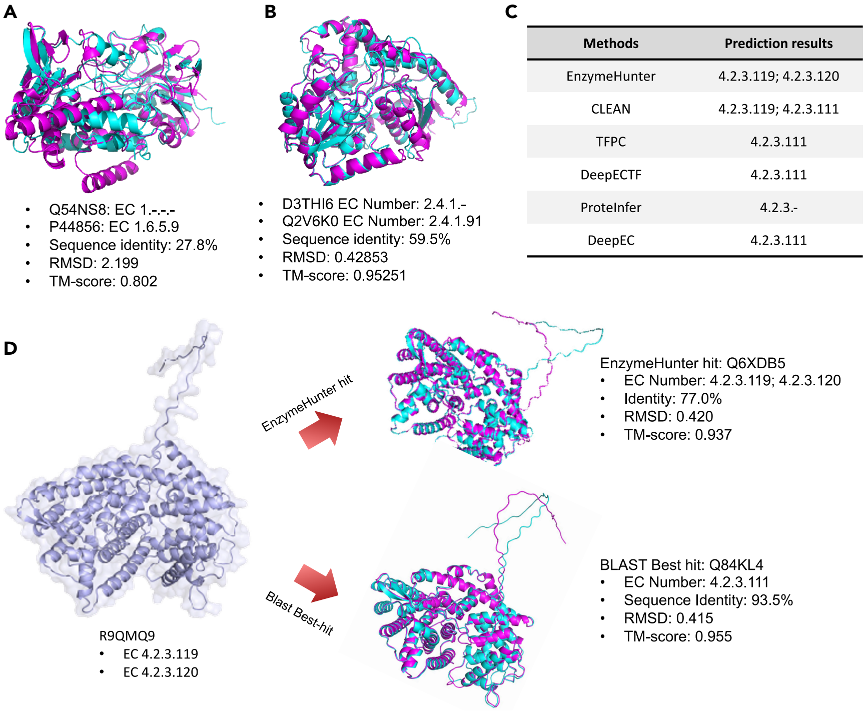

Methods	Prediction results
EnzymeHunter	4.2.3.119; 4.2.3.120
CLEAN	4.2.3.119; 4.2.3.111
TFPC	4.2.3.111
DeepECTF	4.2.3.111
ProteInfer	4.2.3.-
DeepEC	4.2.3.111

Figure 5. Case studies demonstrating EnzymeHunter's ability to resolve ambiguous annotations

(A) Completing a top-level annotation for Q54NS8 (EC 1.-.-.-). EnzymeHunter's prediction of the full EC number (1.6.5.9) is validated by the high structural similarity (TM score = 0.802) to a confirmed enzyme (P44856), despite low sequence identity.

(B) Resolving a partial annotation for D3THI6 (EC 2.4.1.-). The model's correct prediction of the specific function (EC 2.4.1.91) is supported by an exceptionally high structural similarity (TM score = 0.953) to the known enzyme, Q2V6K0.

(C) Table summarizing the predictions from EnzymeHunter and baseline models for the multifunctional enzyme R9QMQ9 (true functions: EC 4.2.3.119 and 4.2.3.120).

(D) Structural basis for correctly identifying multifunctionality. The analysis shows that EnzymeHunter correctly associates the query R9QMQ9 (left) with its true multifunctional structural analog, Q6XDB5 (top, BLAST identity = 77% and TM score = 0.937), while ignoring the misleading high-identity but single-function BLAST hit, Q84KL4 (bottom, BLAST identity = 93.5% and TM score = 0.955).

EnzymeHunter's initial classification of the *Thermus thermophilus* proteome was highly effective. The model demonstrated high confidence, with the predicted probabilities for enzyme vs. non-enzyme forming two distinct clusters (Figure 6B). This accuracy was confirmed on the subset of known enzymes, where a Sankey diagram illustrates that the model correctly identified 213 of 218 annotated proteins (Figure 6C). Having established this high reliability, we then assessed the model's overall functional coverage. As shown in the Venn diagram in Figure 6D, EnzymeHunter not only successfully recovered the vast majority of the 218 known enzymatic functions but, more importantly, assigned putative functions to 617 proteins that were previously uncharacterized. This represents a significant expansion of the annotated enzymome for this organism. To understand the nature of these novel discoveries, we analyzed their functional distribution (Figure 6E). A significant portion of these new annotations were concentrated in specific classes, including the kinase EC 2.7.13.3 and the transporter EC 7.3.2.6, suggesting new insights into the organism's metabolic capabilities and regulatory networks.

The true power of EnzymeHunter, however, lies in its ability to illuminate the functions of previously uncharacterized proteins. A compelling example is the elongation factor thermal unstable (Tu)-A (Q5SHN6), a TrEMBL entry lacking expert annotation. EnzymeHunter confidently assigned it a protein-synthesizing guanosine triphosphatase (GTPase) function (EC 3.6.5.3). This novel prediction was strongly supported by multiple lines of internal and external evidence. Internally, the protein contains all three requisite elongation factor Tu GTP-binding domains (PF00009, PF03144, and PF03143). Externally, despite low sequence identity (33.2%), a structural alignment revealed a high degree of similarity (TM score = 0.815) to a known GTPase, Q975N8 (Figure 6F). In a striking independent validation of our model's predictive power, the UniProt database was subsequently updated in April 2025, assigning this exact EC number (3.6.5.3) to Q5SHN6. This successful "future" prediction provides unequivocal evidence of EnzymeHunter's ability to generate accurate, high-confidence functional hypotheses ahead of manual curation, acting as a powerful engine for discovery.

A second compelling example is the bacterioferritin Q5SI92, another TrEMBL entry lacking functional annotation. EnzymeHunter assigned it a putative ferroxidase activity (EC 1.16.3.1). This prediction is strongly supported by a confluence of evidence: the protein contains the conserved Ferritin domain (PF00210), which is mechanistically linked to the ferroxidase activity required for iron storage, and a retrospective analysis shows that despite low sequence identity (34.4%) to the known ferroxidase Q93PP9, it shares a high degree of structural similarity (TM score = 0.894) (Figure 6G).

Together, these cases exemplify EnzymeHunter's capacity to function as a powerful discovery engine. By generating reliable evidence-backed hypotheses for previously uncharacterized or "hypothetical" proteins, our model can significantly accelerate the annotation of public databases. This is particularly valuable for expanding our understanding of extremophile biochemistry and for the targeted discovery of novel thermostable enzymes with high biotechnological potential.

Learned attention corresponds with known functional sites

To move beyond "black-box" predictions, we computed a composite importance score for each residue by integrating attention scores from the ESM2 model with gradient-based scores from the ResNet101 model. This approach allows us to map the specific sequence-structure features that drive the model's predictions.

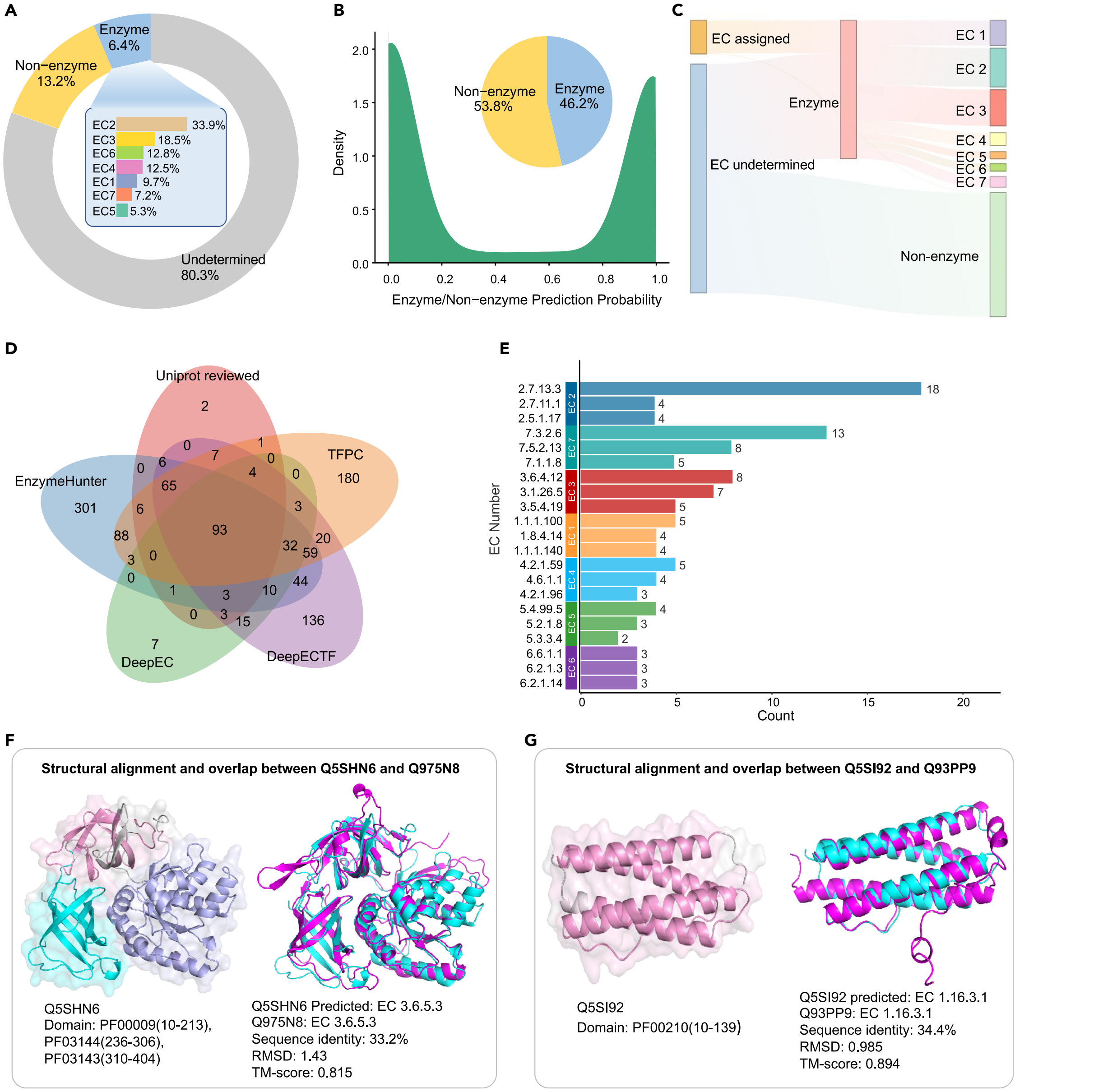

Figure 6. Proteome-wide annotation of *Thermus thermophilus* HB8 reveals EnzymeHunter's discovery capabilities

(A) Distribution of known enzyme classes in the *Thermus thermophilus* proteome.

(B) Histogram of predicted enzyme probabilities shows a clear bimodal distribution, with most proteins confidently classified as either non-enzymes (peak near 0) or enzymes (peak near 1).

(C) Sankey diagram of the proteome-wide prediction results. The diagram visualizes the flow from the total set of 1,795 proteins (left), through the binary enzyme/non-enzyme classification (center), to the final distribution of predicted top-level EC numbers for those classified as enzymes (right).

(D) Venn diagram shows the overlap between proteins identified as enzymes by EnzymeHunter, other models, and the UniProt reviewed set.

(E) Bar chart displays the top three most frequently predicted, full four-digit EC numbers within each of the seven top-level EC categories.

(F) Structural validation for the "future-proof" prediction of elongation factor Tu-A (Q5SHN6) as a GTPase (EC 3.6.5.3), a function later confirmed by UniProt.

(G) Structural validation for the prediction of bacterioferritin (Q5SI92) as a ferroxidase (EC 1.16.3.1).

Our analysis of *o*-succinylbenzoate synthase (OSBS; Q8DJP8, EC 4.2.1.113) provides a clear validation of this framework. As a member of the enolase superfamily, OSBS catalysis depends on the precise coordination of a Mg^{2+} ion to stabilize a reaction intermediate.[28] As visualized in Figure 7A, the residues with the highest importance scores (colored red) form a distinct cluster that directly colocalized with the known active and binding sites (marked with blue stars). This demonstrates that EnzymeHunter has learned to recognize the specific structural environment essential for this function, correctly prioritizing the key residues responsible for metal coordination and substrate stabilization within the catalytic center.

CellPress
OPEN ACCESS

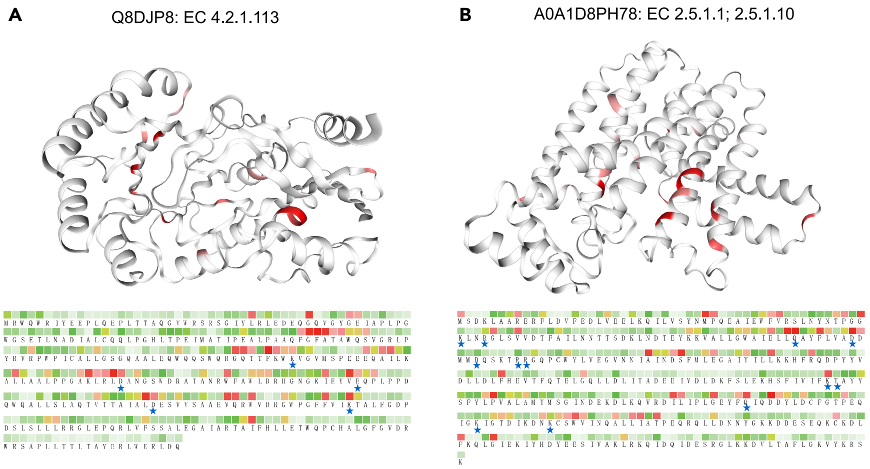

Figure 7. Model interpretability analysis identifies functionally critical residues

Visualization of model-derived importance scores for (A) *o*-succinylbenzoate synthase (Q8DJP8) and (B) farnesyl pyrophosphate synthase (A0A1D8PH78). Residues with the highest scores (red on sequence heatmap and 3D structure) show a strong colocalization with known active and binding sites (blue stars).

The analysis of the multifunctional farnesyl pyrophosphate synthase (FPPS; A0A1D8PH78), which catalyzes two distinct condensation reactions (EC 2.5.1.1 and 2.5.1.10), is even more revealing. This enzyme functions by binding its isoprenyl diphosphate substrates within a large hydrophobic cavity.[29] Crucially, catalysis is mediated by two highly conserved aspartate-rich motifs (DDXX(XX)D) that bind Mg^{2+} ions, which in turn anchor the pyrophosphate groups of the substrates. Our interpretability analysis shows that EnzymeHunter's highest importance scores converge precisely on these essential DDXX(XX)D motifs (Figure 7B). This indicates that the model has learned to identify the core catalytic machinery that is fundamental to the entire class of *trans*-isoprenyl diphosphate synthases. By focusing on this shared critical mechanism, the model can correctly infer both of the enzyme's related functions.

Together, these case studies demonstrate that EnzymeHunter learns a meaningful sequence-structure-function relationship. This ability to autonomously pinpoint functional residues provides strong evidence that its predictions are based on biochemical principles. This interpretability not only builds confidence in the model's outputs but also establishes EnzymeHunter as a powerful tool for generating novel, testable hypotheses about enzyme mechanisms, accelerating the functional characterization of the unannotated proteome.

DISCUSSION

In this study, we have developed EnzymeHunter, a deep-learning framework that demonstrates improved performance in the accuracy and scope of enzyme function prediction. The success of our model is not incremental but is rooted in its synergistic fusion of sequence and structural information, which is fully leveraged by a hierarchically aware contrastive learning framework.

The dual-modal architecture is foundational to EnzymeHunter's ability to operate effectively in challenging scenarios like the low-homology "twilight zone." While protein language models are powerful tools for capturing deep evolutionary context from sequence, functional determination is ultimately a 3D problem. By synergistically integrating a structure-aware representation derived from predicted contact maps, EnzymeHunter gains access to a complementary layer of information about the spatial arrangement of residues that dictates catalysis. This synergy allows the model to build a more holistic understanding of a protein, enabling it to identify true functional analogs even when sequence identity is low. As powerfully illustrated by our case studies, this structure-aware approach provides the necessary context to resolve ambiguities and bypass misleading sequence signals where a sequence-only perspective might falter.

However, the key conceptual advance of our work is the hierarchically aware contrastive learning framework. This is more than a technical modification; it represents a new way of teaching a model the nuanced grammar of the EC system. By explicitly informing the loss function of the EC number's four-level structure, our model learns that functional similarity is not a binary concept. This learned understanding is directly responsible for its exceptional capability in fine-grained prediction. It can resolve the subtle, substrate-level differences at the fourth EC level because its training objective pushes functionally closer enzymes (e.g., sharing three EC digits) to be nearer in the embedding space than more distant ones. Furthermore, this hierarchical knowledge provides a powerful solution to data imbalance. Instead of treating each rare EC number as an isolated, low-data class, our framework allows the model to leverage information from related, more populated classes, enabling robust predictions even for extremely rare enzymes.

The practical implications of these technical advances were demonstrated through our case studies. The successful annotation of the *Thermus thermophilus* proteome, highlighted by the impressive "future" validation of a prediction by a subsequent UniProt update, showcases EnzymeHunter's potential as a real-time discovery engine that can accelerate the slow pace of manual database curation. Moreover, the multifunctional pinene synthase case revealed an elaborated layer of the model's learning. It correctly prioritized a true multifunctional analog over a more structurally similar but functionally inaccurate homolog, demonstrating that the learned embedding space captures a nuanced understanding of function that transcends even global structural similarity. This suggests that the model is not merely a pattern matcher but has learned a more abstract representation of what constitutes a specific catalytic capability.

Finally, our interpretability framework provides a crucial bridge between prediction and biochemical understanding. By showing that the model's learned attention systematically focuses on mechanistically critical residues, such as the primary aspartate-rich motif in farnesyl pyrophosphate synthase, we

demonstrate that EnzymeHunter is not an unexplainable “black box.” This correspondence between the model’s internal logic and established biochemical principles not only builds trust in its predictions but also establishes it as a powerful tool for generating novel, testable hypotheses about enzyme mechanisms and for guiding the experimental characterization of unannotated proteins. To facilitate its practical application and accessibility, the EnzymeHunter web server is publicly available at https://pathoatlas.com/EnzymeHunter (Figure S7), where users can conveniently submit protein sequences for functional prediction.

Despite these advances, we acknowledge certain limitations that pave the way for future work. First, while our ablation studies underscore the potential benefits of incorporating explicit 3D structural data, our primary structure-aware module relies on an ESM-derived 2D contact map to maintain computational efficiency and scalability. Processing high-fidelity 3D coordinates across massive datasets currently incurs prohibitive computational overhead; therefore, developing lightweight architectures capable of efficiently ingesting true 3D structures represents a critical pathway to further boost spatial resolution. Second, translating the continuous representations learned by our hierarchically aware framework into final discrete EC predictions currently relies on empirical thresholding and weighting across different hierarchical levels. Developing robust, data-driven adaptive strategies that dynamically adjust these thresholds based on hierarchical depth or class density remains a key direction for algorithmic optimization. Third, while our model successfully identified a dual-function enzyme, the challenge of predicting the complete functional repertoire of highly promiscuous or “moonlighting” enzymes remains a significant frontier for the field.[30,31] Looking beyond the current EC classification system, a valuable next step is to extend the model’s capabilities from predicting the reaction class to identifying the specific substrates and products involved.[32] The ultimate ambition, however, is to move beyond qualitative functional assignment toward the quantitative prediction of enzyme catalytic efficiency.[33] This would involve regressing key kinetic parameters (such as k_{cat} and K_m), providing a dynamic understanding of an enzyme’s performance that is essential for advancing applications in synthetic biology and metabolic engineering.

In conclusion, by effectively fusing sequence and structural information within a robust, hierarchically aware learning framework, EnzymeHunter provides a powerful, precise, and interpretable solution to some of the most persistent challenges in enzyme function prediction. Its demonstrated ability to perform accurate, fine-grained annotation in low-homology and rare-class scenarios, coupled with its potential as a large-scale discovery tool, positions it to become an indispensable asset for the life sciences community, accelerating the pace of discovery in genomics, metabolic engineering, and drug development.

METHODS

Data curation for model training and evaluation

We constructed distinct datasets for the training and evaluation of EnzymeHunter, our model designed for a two-stage prediction process: (1) binary classification of sequences as enzymes or non-enzymes and (2) multilabel prediction of specific EC numbers.

For the initial enzyme vs. non-enzyme classification, we curated a large-scale dataset from the Swiss-Prot database (release: April 2024). After removing redundant sequences from an initial pool of 573,230 reviewed proteins using CD-HIT (100% sequence identity threshold), we obtained a non-redundant training set of 482,333 proteins. This dataset, comprising a balanced distribution of 235,998 enzymes and 246,335 non-enzymes, was then partitioned. Using stratified random sampling to maintain the enzyme-to-non-enzyme ratio, we split the data into training, validation, and test sets at a ratio of 8:1:1.

For the more complex EC number prediction task, we leveraged the high-fidelity training dataset established by Yu et al.[17] for the CLEAN method. This strategic decision to use a well-curated set of 227,362 expertly reviewed enzyme sequences (from Swiss-Prot, pre-April 2022) covering 5,242 unique EC numbers ensures that our model learns from reliable annotations and allows for direct comparison with the state of the art.

To assess the model’s generalization capabilities, we employed a multitiered evaluation framework. First, for direct benchmarking against established data, we created a combined test set, hereafter referred to as CLEAN-541, by merging the two independent sets from the CLEAN study: the New-392 temporal test set and the Price-149 gold-standard reference set.[34] This combined benchmark comprises 541 unique, high-confidence enzyme sequences. Critically, the ultimate test of a model’s predictive utility is its performance on data generated after its development. We therefore constructed New-1K, a novel, temporally disjoint test set comprising 1,256 protein sequences released in Swiss-Prot after our April 2024 training data cutoff. This challenging set, containing 348 enzymes that span 156 distinct EC numbers and 908 non-enzymes, provides a realistic assessment of EnzymeHunter’s performance on newly discovered proteins.

Hierarchically aware loss function definition

The core objective of our model is to learn protein sequence embeddings that are not only predictive of an enzyme’s function but also reflect the nuanced, hierarchical relationships within the EC classification system. To achieve this, we developed a multicomponent loss function that simultaneously refines the embedding space structure and optimizes prediction accuracy. This total loss, L_{total_loss}, is a weighted sum of a novel dynamic-margin triple loss $L_{triplet}$ and a conventional binary cross-entropy (BCE) loss L_{BCE}:

$$L_{total\_loss} = \lambda L_{triplet} + (1 - \lambda) L_{BCE}, \quad \text{(Equation 1)}$$

where the hyperparameter λ controls the balance between the two underlying objectives.

Our primary innovation lies in the formulation of the triplet loss, $L_{triplet}$, which is designed to model a functionally coherent latent space. The standard triplet loss pushes an anchor embedding (a_i) closer to a positive embedding (p_i) than to a negative one (n_i). However, we posited that the required margin of separation should not be constant but, rather, proportional to the degree of functional divergence between enzymes.

To realize this, we introduce a dynamic margin. The rationale is that a small functional difference (e.g., differing only at the fourth EC digit) should require a smaller separation than a major functional difference (e.g., differing at the first digit). This biologically

"hierarchically aware" margin is integrated into our triplet loss function:

$$L_{triplet} = \sum \max\left(0, d(a_i, p_i) - d(a_i, n_i) + \text{margin}_{dynamic}\right). \quad \text{(Equation 2)}$$

The dynamic margin itself is determined by the hierarchical similarity of the EC numbers of the anchor, positive, and negative samples:

$$\text{margin}_{dynamic} = \text{margin}_{base} \times \left(2 - S\left(EC_{a_i}, EC_{p_i}\right) + S\left(EC_{a_i}, EC_{n_i}\right)\right). \quad \text{(Equation 3)}$$

The similarity score $S(EC_i,EC_j)$ is a weighted sum of matching EC digits across the four levels of the hierarchy (l = 1,2,3,4), with weights w_l = [0.4, 0.3, 0.2, 0.1] prioritizing higher-level similarity. The calculation captures the EC system's structure by halting the summation at the first point of divergence:

$$S\left(EC_i, EC_j\right) = \max\left(\sum_{l=1}^{4} w_l * I\left(EC_{il}, EC_{jl}\right)\right). \quad \text{(Equation 4)}$$

While the triplet loss structures the embedding space, the binary cross-entropy component, L_{BCE}, ensures the model's direct predictive power. We apply a standard BCE loss to the outputs for each of the three enzymes in a triplet, comparing the predicted probability distribution (p_j) against the ground-truth label vector (t_j):

$$L_{BCE} = \sum_{j \in (a_i, p_i, n_i)} L_{base_{BCE}}\left(t_j, p_j\right). \quad \text{(Equation 5)}$$

Together, these two loss components compel our model to learn functionally rich representations that are both hierarchically structured and highly predictive, addressing a key challenge in computational enzymology.

Model training, inference, and prediction refinement

The training of EnzymeHunter is centered on a triplet-based contrastive learning framework, designed to shape a functionally meaningful embedding space. To maximize the efficacy of this training, we move beyond random sampling and implement a hard negative mining strategy.[35] The rationale behind this approach is to present the model with more challenging and informative training signals. Specifically, negative samples—enzymes with different EC numbers—are preferentially selected if they are located proximally to the anchor protein within the EC hierarchy space, compelling the model to learn the fine-grained distinctions between functionally related yet distinct enzymes.

A key challenge in this framework is the existence of singleton EC numbers (occurrence count = 1), which lack natural positive pairs for triplet construction. To ensure these rare but biologically important enzymes contribute to training, we generate synthetic positive samples by injecting adaptively scaled Gaussian noise into their feature vectors. Crucially, the magnitude of the injected noise is adaptively scaled by the standard deviation of the original feature vector, thereby creating biologically plausible variations while preserving core functional characteristics. This procedure is applied for ten augmentation iterations (i) to both the 2,560-dimensional ESM2 embedding (E_p) and the 2,048-dimensional contact map embedding (C_p) of a given protein (p), and the resulting augmented features ($E'_{p,i}$ and $C'_{p,i}$) are then concatenated. The process is formulated as

$$E'_{p,i} = E_p + \text{Randn}_{\text{like}\left(E_p\right)} * \text{Std}\left(E_p\right) * 0.01, \quad \text{(Equation 6)}$$

$$C'_{p,i} = C_p + \text{Randn}_{\text{like}\left(C_p\right)} * \text{Std}\left(C_p\right) * 0.01, \quad \text{(Equation 7)}$$

$$M_{p,i} = \left[E'_{p,i};\ C'_{p,i}\right]. \quad \text{(Equation 8)}$$

Following the generation of a 128-dimensional embedding for a query enzyme, the final EC number assignment is determined by a hierarchical decision process. The primary prediction is made using a maximum separation algorithm, as introduced by Yu et al.,[17] which analyzes the distribution of distances to the top candidate functional centroids.

The algorithm first identifies the ten EC functional centroids with the smallest Euclidean distance (d_i) to the query embedding. It then analyzes the "gap" in this distance distribution to find a natural cutoff point. To achieve this, it calculates the deviation of each distance from the mean (q_i) and the separation between consecutive deviations (g_i). The initial decision follows a cutoff rule: call candidates EC_i whose separation g_i exceeds the average separation $\overline{g}$ are included in the preliminary prediction set.

$$q_i = |d_i - \overline{d}| = \left|d_i - \frac{1}{10}\sum_{i=1}^{10} d_i\right|, i \in [1, 10], \quad \text{(Equation 9)}$$

$$g_i = |q_i - q_{i-1}|, i \in [2, 10], \quad \text{(Equation 10)}$$

$$\overline{g} = \frac{1}{9}\sum_{i=2}^{10} g_i, i \in [2, 10]. \quad \text{(Equation 11)}$$

If this initial step yields no candidates, a global high-confidence homology refinement step is triggered. In this stage, we perform a comprehensive search for the query protein against all reference enzymes in our training set database using DIAMOND[36] and TM-Vec,[37] strictly excluding any test samples to prevent information leakage. If any reference enzyme is found that meets a stringent dual-similarity threshold (>90% sequence identity and a TM score >0.9), the EC number of that reference enzyme is assigned as a high-confidence prediction.

If both the maximum separation and the global homology refinement steps fail to produce a prediction, a final default rule is applied, assigning only the top-ranked (closest) candidate, EC_1, from the initial distance calculation. This multilayered strategy ensures that predictions are made with the highest possible confidence, intelligently balancing statistical evidence from the embedding space with direct, high-confidence homology.

Model performance evaluation

To comprehensively evaluate the model's performance on the multilabel task of EC number prediction, we employed a suite of standard classification metrics. A key challenge in this domain is the significant class imbalance inherent in enzyme functional annotations, where certain EC classes are far more prevalent than others. To address this, we calculated precision, recall,

and F1 score using a weighted averaging strategy, as well as MCC. This approach computes the metric independently for each class and then calculates an average, where each class's score is weighted by its support (the number of true instances for that class). This ensures that the evaluation properly reflects the model's performance across the entire dataset, taking the class distribution into account. Overall accuracy was also computed.

All metrics were calculated using the Python scikit-learn package. The specific formulations for the weighted metrics are as follows:

$$\text{Precision}_{\text{weight}} = \sum_{i=1}^{C} (w_i * \text{Precision}_i) = \sum_{i=1}^{C} \left(w_i * \frac{TP_i}{TP_i + FP_i} \right), \quad \text{(Equation 12)}$$

$$\text{Recall}_{\text{weight}} = \sum_{i=1}^{C} (w_i * \text{Recall}_i) = \sum_{i=1}^{C} \left(w_i * \frac{TP_i}{TP_i + FN_i} \right), \quad \text{(Equation 13)}$$

$$F1_{\text{weight}} = \sum_{i=1}^{C} (w_i * F1_i) = \sum_{i=1}^{C} \left(w_i * 2 * \frac{\text{Precision}_i * \text{Recall}_i}{\text{Precision}_i + \text{Recall}_i} \right), \quad \text{(Equation 14)}$$

$$MCC = \sum_{i=1}^{C} \left(\frac{TP_i \times TN_i - FP_i \times FN_i}{\sqrt{(+FP_i)(TP_i + FN_i)(TN_i + FP_i)(TN_i + FN_i)}} \right), \quad \text{(Equation 15)}$$

where

- C is the total number of EC classes;
- for class i, TP_i, FP_i, and FN_i are the counts of the true positives, false positives, and false negatives, respectively; and
- w_i is the weight of class i, defined as the proportion of samples belonging to class i in the dataset.

Configuration of custom CNN module for ablation study

Specifically, a custom three-layer CNN (3× CNN) was designed to process single-channel binary inputs. The architecture begins with an initial 7 × 7 convolutional layer (stride = 8), which utilizes a large receptive field for rapid spatial downsampling and preliminary feature extraction. This is followed by two 1 × 1 convolutional layers (stride = 2) to further compress the spatial resolution while expanding the feature dimensionality from 32 to 128 channels. Each convolutional operation is followed by batch normalization and a ReLU activation function. Finally, the network employs an adaptive global average pooling layer and a fully connected layer to output a fixed 256-dimensional feature vector.

RESOURCE AVAILABILITY

Lead contact

Requests for further information and resources should be directed to and will be fulfilled by the lead contact, Prof. Jun Wu (jwu@bio.ecnu.edu.cn).

Materials availability

This study did not generate new unique reagents.

Data and code availability

The EnzymeHunter model datasets and code are available via GitHub at https://github.com/cgxbio/EnzymeHunter and via Zenodo at https://zenodo.org/records/18896678.[38] The original protein sequence training and testing data can be downloaded from the UniProt database (https://www.uniprot.org/), and the New-392 and Price-149 datasets can be downloaded from CLEAN (https://github.com/tttianhao/CLEAN). All PDB files are obtained from the AlphaFold Protein Structure Database https://alphafold.com/, and if no PDB file is available, predictions from AlphaFold3 (https://alphafoldserver.com/) or ESMFold (https://github.com/facebookresearch/esm) are used. A web version of EnzymeHunter is accessible at https://pathoatlas.com/EnzymeHunter.

ACKNOWLEDGMENTS

This work was supported by the National Key Research and Development Program of China (2023YFC2706503), the National Natural Science Foundation of China (32370720), the Natural Science Foundation of Shanghai 2025 (25ZR1401106), the Open Research Fund of Key Laboratory of Advanced Theory and Application in Statistics and Data Science – MOE, ECNU, and the Open Research Fund of Key Laboratory of MEA, Ministry of Education, ECNU.

AUTHOR CONTRIBUTIONS

J.W. and T.S. conceived and designed the study; G.C. and J.W. developed the model; G.C., J.W., J.O., X.X., C.L., Y.Z., and S.Y. performed data analysis and visualization; J.O. and X.X. built the web server; G.C. and J.W. wrote the manuscript; and J.W. and T.S. revised the manuscript. All authors read and approved the final manuscript.

DECLARATION OF INTERESTS

The authors declare no competing interests.

SUPPLEMENTAL INFORMATION

Supplemental information can be found online at https://doi.org/10.1016/j.patter.2026.101567.

Received: December 10, 2025
Revised: March 16, 2026
Accepted: April 28, 2026
Published: May 28, 2026

REFERENCES

1. Agarwal, P.K. (2006). Enzymes: An integrated view of structure, dynamics and function. Microb. Cell Fact. *5*, 2. https://doi.org/10.1186/1475-2859-5-2.
2. Makrydaki, E., Donini, R., Krueger, A., Royle, K., Moya Ramirez, I., Kuntz, D.A., Rose, D.R., Haslam, S.M., Polizzi, K.M., and Kontoravdi, C. (2024). Immobilized enzyme cascade for targeted glycosylation. Nat. Chem. Biol. *20*, 732–741. https://doi.org/10.1038/s41589-023-01539-4.
3. UniProt Consortium (2025). UniProt: the Universal Protein Knowledgebase in 2025. Nucleic Acids Res. *53*, D609–D617. https://doi.org/10.1093/nar/gkae1010.
4. Wu, J., Hu, Y., Perlin, M.H., Danko, D., Lu, J., Oliveira, M., Werner, J., Zambrano, M.M., Sierra, M.A., Osuolale, O.O., et al. (2024). Landscape of global urban environmental resistome and its association with local socioeconomic and medical status. Sci. China Life Sci. *67*, 1292–1301. https://doi.org/10.1007/s11427-023-2504-1.
5. Santos, C.A., Morais, M.A.B., Mandelli, F., Lima, E.A., Miyamoto, R.Y., Higasi, P.M.R., Araujo, E.A., Paixão, D.A.A., Junior, J.M., Motta, M.L., et al. (2025). A metagenomic 'dark matter' enzyme catalyses oxidative cellulose conversion. Nature *639*, 1076–1083. https://doi.org/10.1038/s41586-024-08553-z.

CellPress
OPEN ACCESS

6. Osburn, E.D., Mcbride, S.G., and Strickland, M.S. (2024). Microbial dark matter could add uncertainties to metagenomic trait estimations. Nat. Microbiol. *9*, 1427–1430. https://doi.org/10.1038/s41564-024-01687-w.
7. Bairoch, A. (2000). The ENZYME database in 2000. Nucleic Acids Res. *28*, 304–305. https://doi.org/10.1093/nar/28.1.304.
8. McDonald, A.G., and Tipton, K.F. (2023). Enzyme nomenclature and classification: the state of the art. FEBS J. *290*, 2214–2231. https://doi.org/10.1111/febs.16274.
9. Jacobson, M.P., Kalyanaraman, C., Zhao, S., and Tian, B. (2014). Leveraging structure for enzyme function prediction: methods, opportunities, and challenges. Trends Biochem. Sci. *39*, 363–371. https://doi.org/10.1016/j.tibs.2014.05.006.
10. Ward, R.M., Venner, E., Daines, B., Murray, S., Erdin, S., Kristensen, D.M., and Lichtarge, O. (2009). Evolutionary Trace Annotation Server: automated enzyme function prediction in protein structures using 3D templates. Bioinformatics *25*, 1426–1427. https://doi.org/10.1093/bioinformatics/btp160.
11. Kim, H.R., Ji, H., Kim, G.B., and Lee, S.Y. (2025). Enzyme functional classification using artificial intelligence. Trends Biotechnol. *43*, 2214–2231. https://doi.org/10.1016/j.tibtech.2025.03.003.
12. Wang, W., and Huang, X. (2025). AI empowers exploration of protein universe. Sci. China Life Sci. *68*, 588–589. https://doi.org/10.1007/s11427-024-2673-9.
13. Wang, D., Tan, Z., Gao, J., Zhang, S., Shen, J., and Lu, Y. (2025). AI4Protein: transforming the future of protein design. Sci. China Life Sci. *68*, 2880–2890. https://doi.org/10.1007/s11427-024-2906-3.
14. Rives, A., Meier, J., Sercu, T., Goyal, S., Lin, Z., Liu, J., Guo, D., Ott, M., Zitnick, C.L., Ma, J., and Fergus, R. (2021). Biological structure and function emerge from scaling unsupervised learning to 250 million protein sequences. Proc. Natl. Acad. Sci. USA *118*, e2016239118. https://doi.org/10.1073/pnas.2016239118.
15. Ryu, J.Y., Kim, H.U., and Lee, S.Y. (2019). Deep learning enables high-quality and high-throughput prediction of enzyme commission numbers. Proc. Natl. Acad. Sci. USA *116*, 13996–14001. https://doi.org/10.1073/pnas.1821905116.
16. Kim, G.B., Kim, J.Y., Lee, J.A., Norsigian, C.J., Palsson, B.O., and Lee, S.Y. (2023). Functional annotation of enzyme-encoding genes using deep learning with transformer layers. Nat. Commun. *14*, 7370. https://doi.org/10.1038/s41467-023-43216-z.
17. Yu, T., Cui, H., Li, J.C., Luo, Y., Jiang, G., and Zhao, H. (2023). Enzyme function prediction using contrastive learning. Science *379*, 1358–1363. https://doi.org/10.1126/science.adf2465.
18. Qian, H., Wang, Y., Zhou, X., Gu, T., Wang, H., Lyu, H., Li, Z., Li, X., Zhou, H., Guo, C., et al. (2025). ESM-Ezy: a deep learning strategy for the mining of novel multicopper oxidases with superior properties. Nat. Commun. *16*, 3274. https://doi.org/10.1038/s41467-025-58521-y.
19. Van Der Weg, K., Merdivan, E., Piraud, M., and Gohlke, H. (2025). TopEC: prediction of Enzyme Commission classes by 3D graph neural networks and localized 3D protein descriptor. Nat. Commun. *16*, 2737. https://doi.org/10.1038/s41467-025-57324-5.
20. Douglas, J., Carter, C.W., Jr., and Wills, P.R. (2024). HetMM: A Michaelis-Menten model for non-homogeneous enzyme mixtures. iScience *27*, 108977. https://doi.org/10.1016/j.isci.2024.108977.
21. Zhang, S., Zhang, T., and Fu, Y. (2023). Proteome-wide structural analysis quantifies structural conservation across distant species. Genome Res. *33*, 1975–1993. https://doi.org/10.1101/gr.277771.123.
22. Kabir, M.N., and Wong, L. (2022). EnsembleFam: towards more accurate protein family prediction in the twilight zone. BMC Bioinf. *23*, 90. https://doi.org/10.1186/s12859-022-04626-w.
23. He, K.M., Zhang, X.Y., Ren, S.Q., and Sun, J. (2016). Deep residual learning for image recognition. In 2016 IEEE Conference on Computer Vision and Pattern Recognition (CVPR) (IEEE), pp. 770–778. https://doi.org/10.1109/CVPR.2016.90.
24. Sanderson, T., Bileschi, M.L., Belanger, D., and Colwell, L.J. (2023). ProteInfer, deep neural networks for protein functional inference. eLife *12*, e80942. https://doi.org/10.7554/elife.80942.
25. Buton, N., Coste, F., and Le Cunff, Y. (2023). Predicting enzymatic function of protein sequences with attention. Bioinformatics *39*, btad620. https://doi.org/10.1093/bioinformatics/btad620.
26. Varadi, M., Anyango, S., Deshpande, M., Nair, S., Natassia, C., Yordanova, G., Yuan, D., Stroe, O., Wood, G., Laydon, A., et al. (2022). AlphaFold Protein Structure Database: massively expanding the structural coverage of protein-sequence space with high-accuracy models. Nucleic Acids Res. *50*, D439–D444. https://doi.org/10.1093/nar/gkab1061.
27. Lin, Z., Akin, H., Rao, R., Hie, B., Zhu, Z., Lu, W., Smetanin, N., Verkuil, R., Kabeli, O., Shmueli, Y., et al. (2023). Evolutionary-scale prediction of atomic-level protein structure with a language model. Science *379*, 1123–1130. https://doi.org/10.1126/science.ade2574.
28. Klenchin, V.A., Ringia, E.A.T., Gerlt, J.A., and Rayment, I. (2003). Evolution of enzymatic activity in the enolase superfamily: Structural and mutagenic studies of the mechanism of the reaction catalyzed by succinylbenzoate synthase from. Biochemistry *42*, 14427–14433. https://doi.org/10.1021/bi035545v.
29. Tarshis, L.C., Yan, M., Poulter, C.D., and Sacchettini, J.C. (1994). Crystal structure of recombinant farnesyl diphosphate synthase at 2.6-A resolution. Biochemistry *33*, 10871–10877. https://doi.org/10.1021/bi00202a004.
30. Van der Knaap, J.A., and Verrijzer, C.P. (2025). Moonlighting Enzymes at the Interface Between Metabolism and Epigenetics. Annu. Rev. Biochem. *94*, 279–303. https://doi.org/10.1146/annurev-biochem-030122-044718.
31. Guo, D., Meng, Y., Zhao, G., Wu, Q., and Lu, Z. (2025). Moonlighting functions of glucose metabolic enzymes and metabolites in cancer. Nat. Rev. Cancer *25*, 426–446. https://doi.org/10.1038/s41568-025-00800-3.
32. Shepherd, R.A., Fihn, C.A., Tabag, A.J., Mckinnie, S.M.K., and Sanchez, L.M. (2025). 'Need for speed: high throughput' - mass spectrometry approaches for high-throughput directed evolution screening of natural product enzymes. Nat. Prod. Rep. *42*, 1037–1054. https://doi.org/10.1039/d4np00053f.
33. Wang, Z., Xie, D., Wu, D., Luo, X., Wang, S., Li, Y., Yang, Y., Li, W., and Zheng, L. (2025). Robust enzyme discovery and engineering with deep learning using CataPro. Nat. Commun. *16*, 2736. https://doi.org/10.1038/s41467-025-58038-4.
34. Price, M.N., Wetmore, K.M., Waters, R.J., Callaghan, M., Ray, J., Liu, H., Kuehl, J.V., Melnyk, R.A., Lamson, J.S., Suh, Y., et al. (2018). Mutant phenotypes for thousands of bacterial genes of unknown function. Nature *557*, 503–509. https://doi.org/10.1038/s41586-018-0124-0.
35. Robinson, J., Chuang, C.-Y., Sra, S., and Jegelka, S. (2020). Contrastive learning with hard negative samples. arXiv. https://doi.org/10.48550/arXiv.2010.04592.
36. Buchfink, B., Xie, C., and Huson, D.H. (2015). Fast and sensitive protein alignment using DIAMOND. Nat. Methods *12*, 59–60. https://doi.org/10.1038/nmeth.3176.
37. Hamamsy, T., Morton, J.T., Blackwell, R., Berenberg, D., Carriero, N., Gligorijevic, V., Strauss, C.E.M., Leman, J.K., Cho, K., and Bonneau, R. (2024). Protein remote homology detection and structural alignment using deep learning. Nat. Biotechnol. *42*, 975–985. https://doi.org/10.1038/s41587-023-01917-2.
38. Cao, G., Ouyang, J., Xiong, X., Liu, C., Zhang, Y., Yang, S., Shi, T., and Wu, J. (2026). EnzymeHunter: Achieving fine-grained enzyme function prediction with a hierarchically-aware contrastive learning framework. Zenodo. https://zenodo.org/records/18896678.

Patterns, Volume *7*

Supplemental information

EnzymeHunter: Achieving fine-grained enzyme function prediction with a hierarchically aware contrastive learning framework

Guoxin Cao, Jian Ouyang, Xiangyi Xiong, Changle Liu, Yi Zhang, Siqi Yang, Tieliu Shi, and Jun Wu

Supplemental information

EnzymeHunter: Achieving fine-grained enzyme function prediction with a hierarchically-aware contrastive learning framework

Guoxin Cao, Jian Ouyang, Xiangyi Xiong, Changle Liu, Yi Zhang, Siqi Yang, Tieliu Shi, Jun Wu

Supplemental Figures

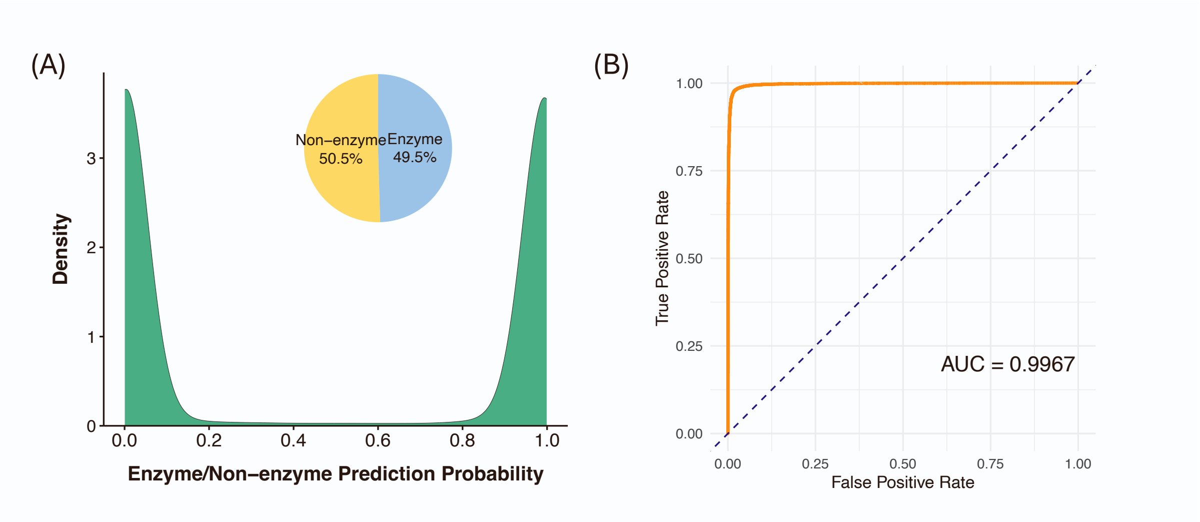

Figure S1. Bimodal distribution of enzyme prediction probabilities and performance in enzyme classification. (A) The histogram of predicted enzyme probabilities shows a clear bimodal distribution, with most proteins confidently classified as either non-enzymes (peak near 0) or enzymes (peak near 1). (B) Performance on the binary task of distinguishing enzymes from non-enzymes.

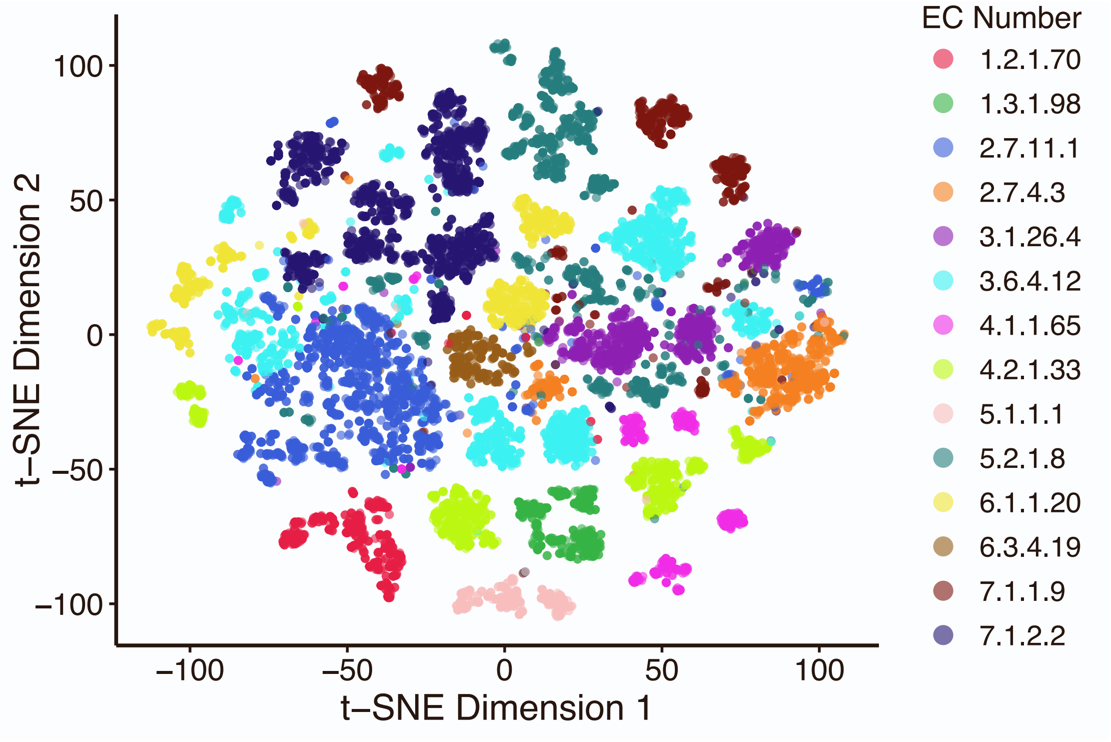

Figure S2. t-SNE projection of combined ESM2 and ResNet embedding representations. t-SNE projection of the concatenated 2560-dimensional ESM2 embeddings and 2048-dimensional ResNet embeddings.

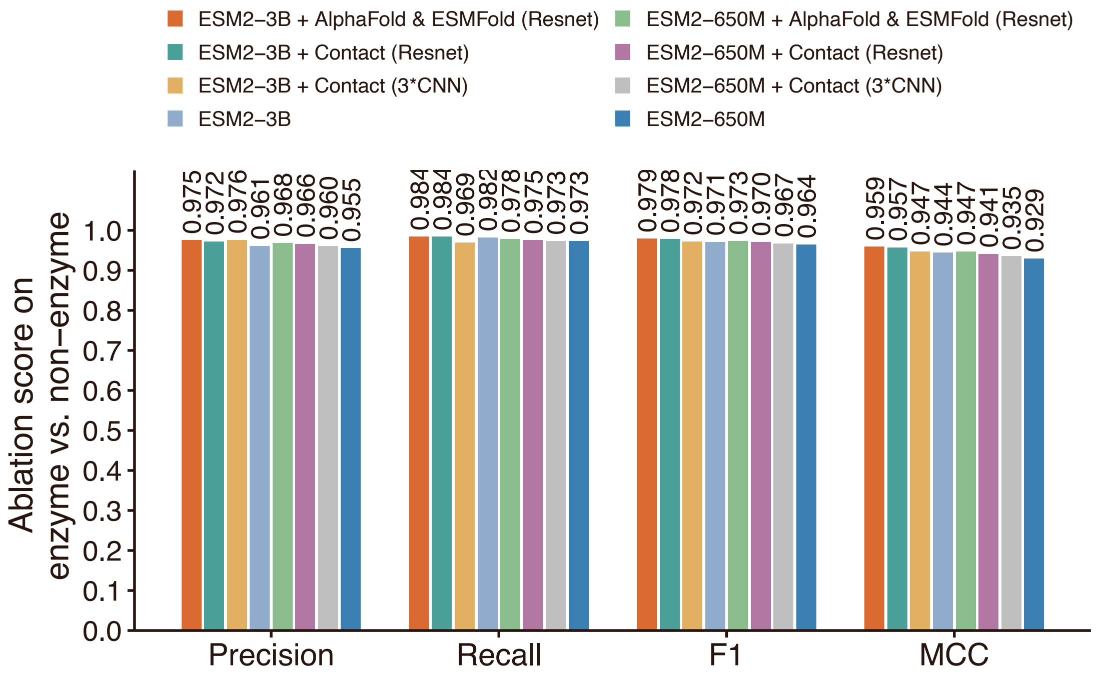

Figure S3. Performance comparison of different feature combinations on the enzyme vs. non-enzyme dataset. The model performance is evaluated using Precision, Recall, F1 score, and MCC.

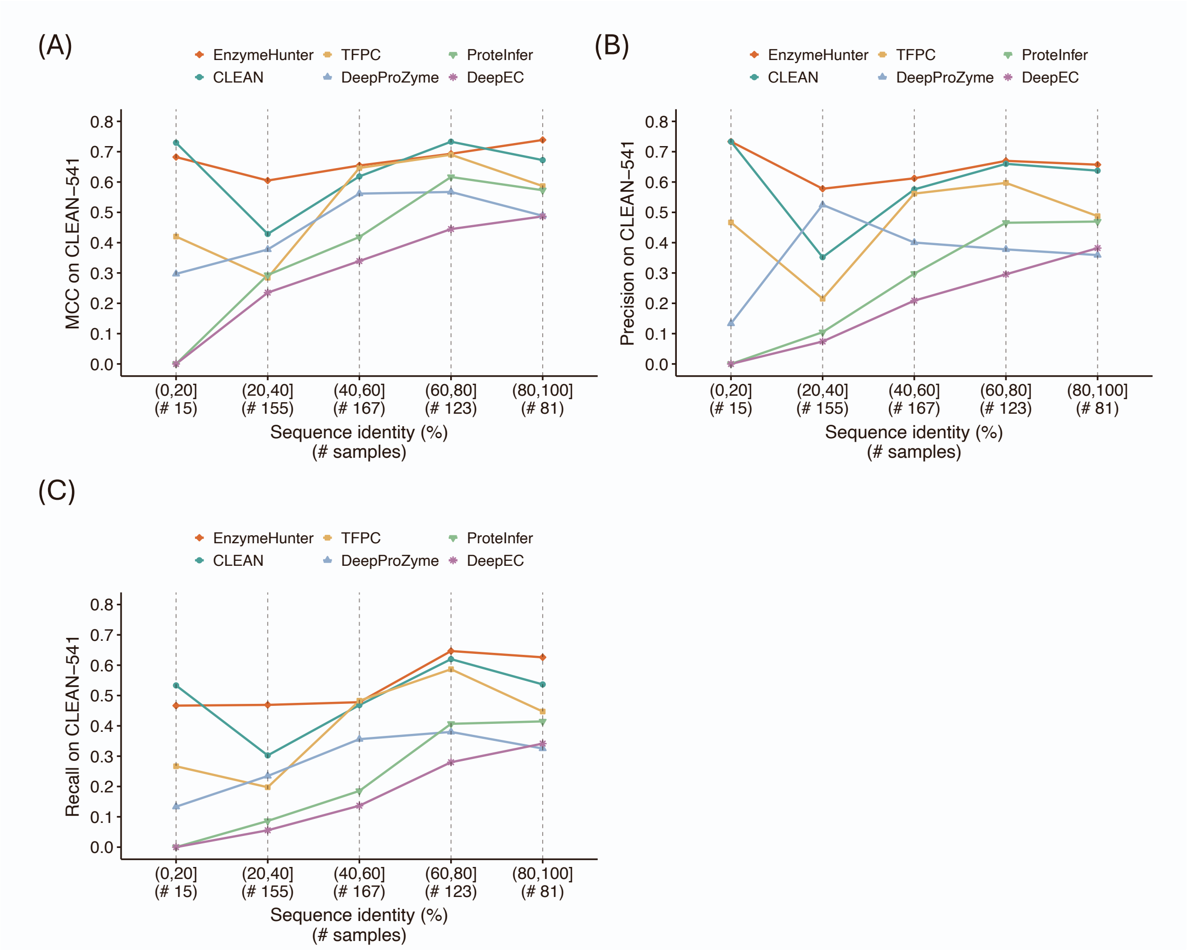

Figure S4. The performance results of the model on the Sequence identity in the CLEAN-541 dataset. (A) The results of the MCC metric on the CLEAN-541 dataset. (B) The results of the Precision metric on the CLEAN-541 dataset. (C) The results of the Recall metric on the CLEAN-541 dataset.

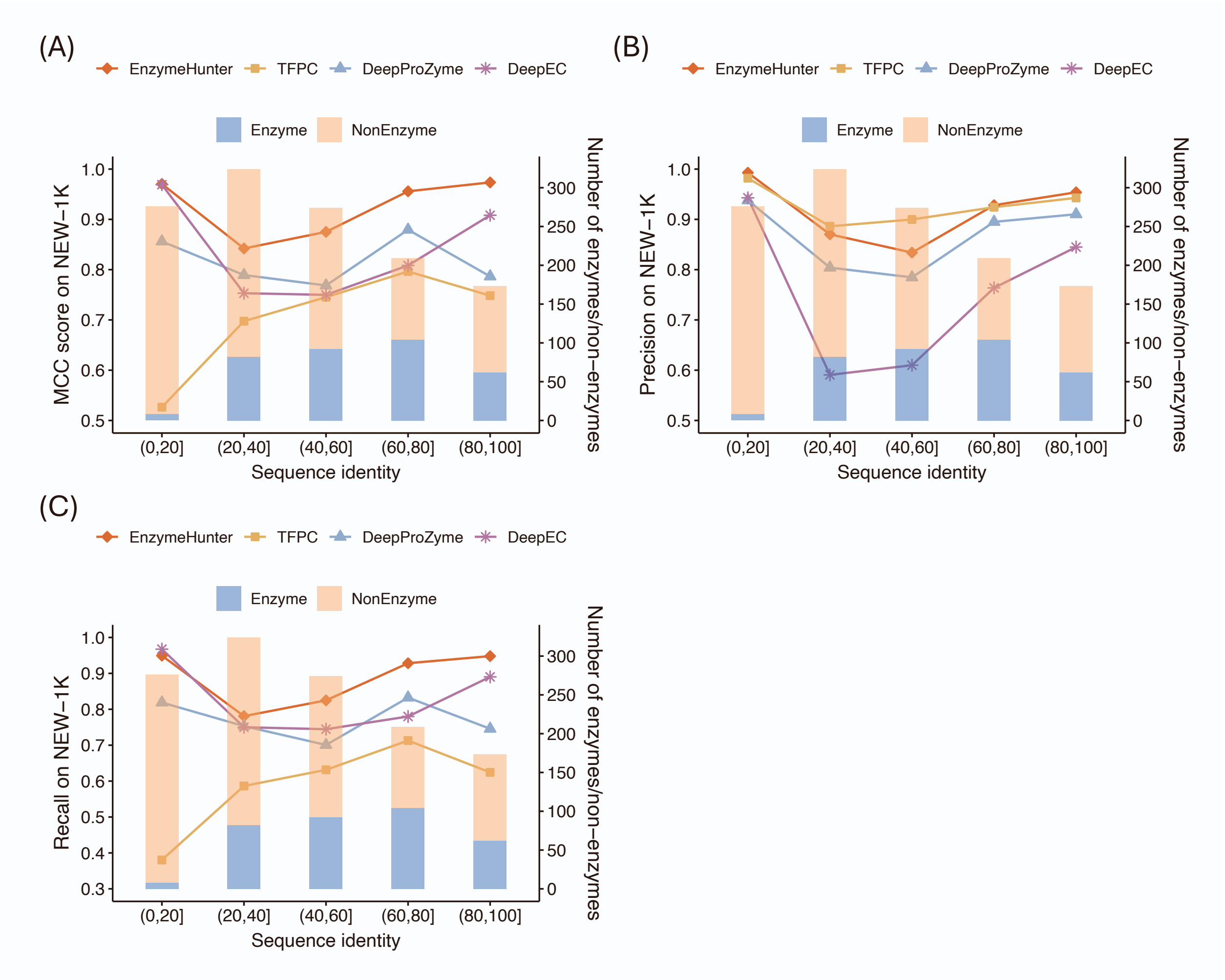

Figure S5. The model performance results on the NEW-1K dataset. (A) The results of the MCC metric on the NEW-1K dataset. (B) The results of the Precision metric on the NEW-1K dataset. (C) The results of the Recall metric on the NEW-1K dataset.

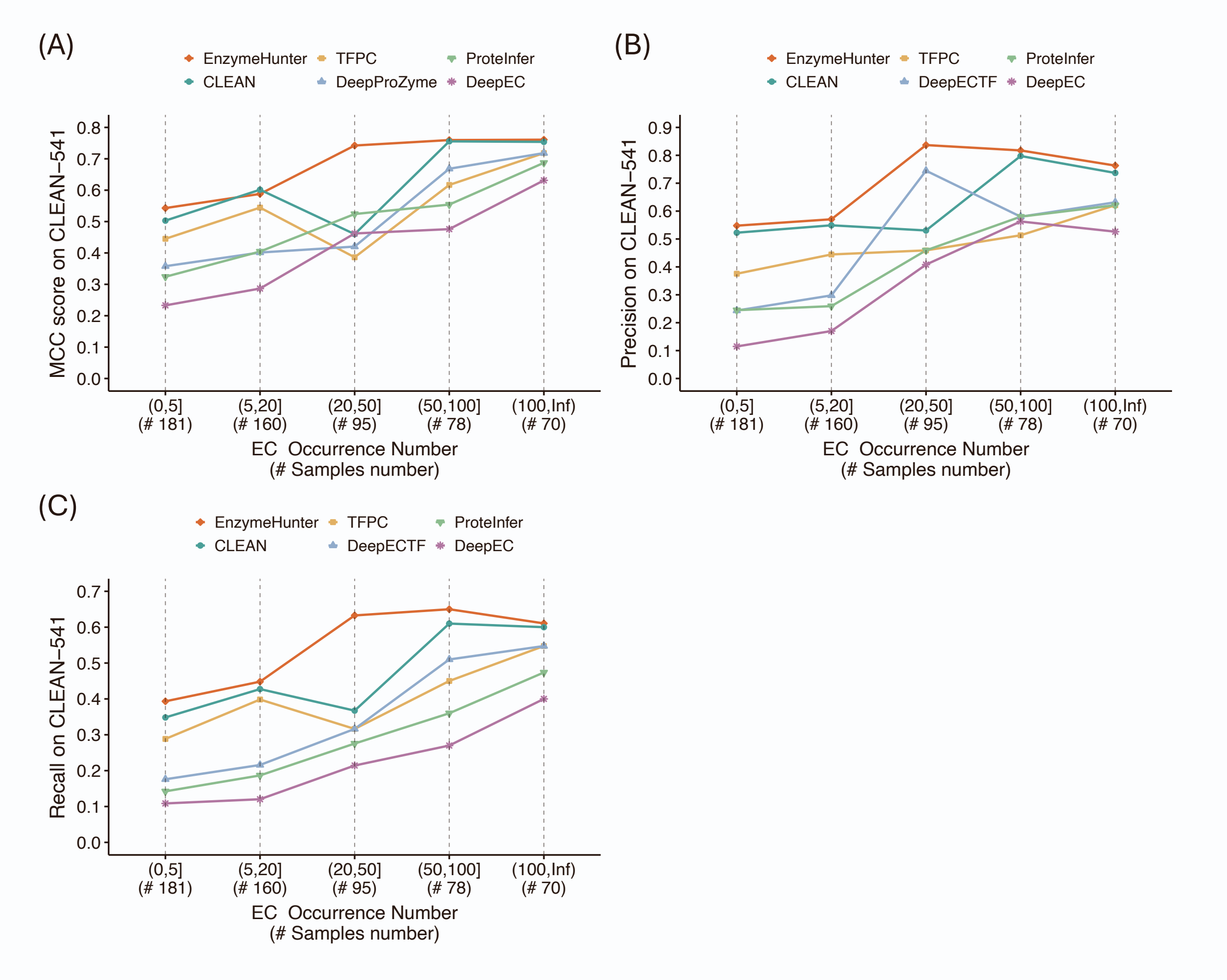

Figure S6. The performance results of the model on the EC Occurrence Number in the CLEAN-541 dataset. (A) The results of the MCC metric on the CLEAN-541 dataset. (B) The results of the Precision metric on the CLEAN-541 dataset. (C) The results of the Recall metric on the CLEAN-541 dataset.

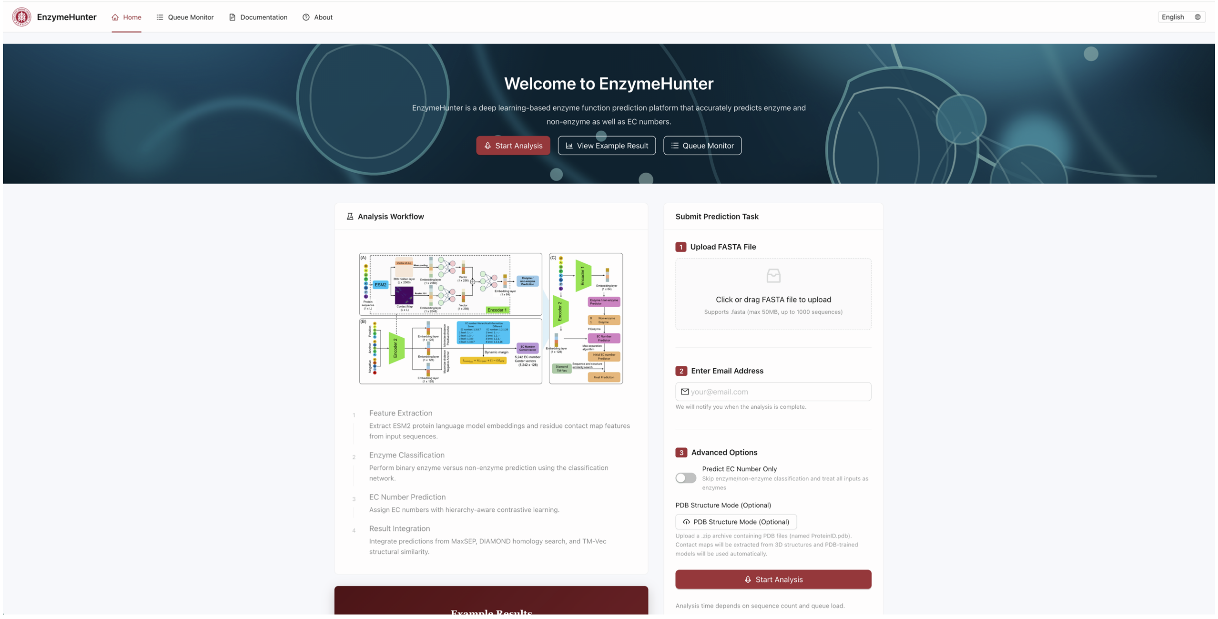

Figure S7. The web interface of EnzymeHunter (https://pathoatlas.com/EnzymeHunter). Users can upload FASTA files of amino acid sequences to obtain enzyme function prediction results.
